# HAP-SAMPLE2: data-based resampling for association studies with admixture

**DOI:** 10.1093/bioinformatics/btaf333

**Published:** 2025-06-13

**Authors:** George Sun, Bryan W Ting, Fred A Wright, Yi-Hui Zhou

**Affiliations:** Bioinformatics Research Center, North Carolina State University, Raleigh, NC 27695, United States; Bioinformatics Research Center, North Carolina State University, Raleigh, NC 27695, United States; Bioinformatics Research Center, North Carolina State University, Raleigh, NC 27695, United States; Department of Biological Sciences, North Carolina State University, Raleigh, NC 27695, United States; Department of Statistics, North Carolina State University, Raleigh, NC 27695, United States; Bioinformatics Research Center, North Carolina State University, Raleigh, NC 27695, United States; Department of Biological Sciences, North Carolina State University, Raleigh, NC 27695, United States; Department of Statistics, North Carolina State University, Raleigh, NC 27695, United States

## Abstract

**Motivation:**

HAP-SAMPLE2 extends the functionality of the original HAP-SAMPLE tool for simulating genotype-phenotype data, now with features to handle population admixture and rare variant analysis. It allows users to define parameters such as disease prevalence and allele effect sizes for both common and rare variant simulations.

**Results:**

HAP-SAMPLE2 provides an efficient means for simulating complex datasets, suitable for large-scale projects like the 1000 Genomes Project. Its capabilities for population admixture allow users to create admixed populations or preserve substructures while introducing novel variation through artificial recombination. Additionally, the tool supports burden testing for rare variants using fixed and Madsen-Browning weighting schemes.

**Availability and implementation:**

The software, along with a detailed vignette, is available on GitHub: https://github.com/M3dical/HAPSAMPLE2.

## 1 Introduction

Recent advancements in statistical genetics have increased the prominence of simulation methods as valuable tools for complementing genotype-phenotype association studies (Wright *et al.* 2007, Wharrie *et al.* 2023, Jarquin *et al.* 2024, Schraiber and Edge 2024). Simulated datasets are instrumental in performing power analyses and evaluating various statistical learning methods (Yuan *et al.* 2012, Zhou *et al.* 2018). In this work, we introduce HAP-SAMPLE2, an extension of the methodologies previously established in HAP-SAMPLE (Wright *et al.* 2007). HAP-SAMPLE2 is designed to simulate datasets for admixed populations by resampling from real datasets, utilizing either common or rare variants, and generating phenotypes suitable for both case-control and quantitative trait study designs. Over the years, numerous software packages have been developed to facilitate dataset simulations (Peng *et al.* 2013).

For instance, HAPSIMU (Zhang *et al.* 2008) is a C++ simulation platform for association studies that incorporates HapMap data and recombination rates. It supports both qualitative and quantitative data under continuous migration or discrete models, simulating both genotype and phenotype data.

HAPGEN2 (Su *et al.* 2011), the successor to HAPGEN, is a C++ tool tailored for simulating case-control disease datasets, allowing for multiple disease loci on a single chromosome. It accounts for Single Nucleotide Polymorphism (SNP)-level recombination rates and enables the specification of heterozygote and homozygote relative risks, providing simulated haplotype and genotype data for cases and controls.

Hapnest (Wharrie *et al.* 2023) uses a combination of real genotypes and coalescent-based genotype simulation and can simulate binary and quantitative phenotypes. The binary simulations are not performed consistently with typical logistic modeling, and the package is optimized for polygenic risk score simulation. Although admixed samples can be generated, the authors point out that the approach does not correspond to standard genetic admixtures.

Similarly, simuPOP (Peng and Kimmel 2005), alongside its companion tool simuGWAS, is a Python-based framework that leverages HapMap data, including fine-scale recombination rates, to model population evolution. It can simulate both forward and backward evolution, with the capability to handle gene-environment interaction models through user-specified markers. Although powerful, simuPOP relies on the user to write Python scripts to integrate its various components (Peng and Amos 2010), while simuGWAS further facilitates simulations and post-processing for association studies.

GWAsimulator (Li and Li 2008), a C++ program available across Linux, Windows, and Mac platforms, uses a “rapid window algorithm” to simulate case-control or population samples, assuming Hardy-Weinberg equilibrium. This approach simulates recombination by sampling contiguous SNPs in a Markovian fashion and supports user-specified multi-locus disease models with the potential for two-way interactions.

SEQSIMLA and SEQSIMLA2 (Ren‐Hua *et al.* 2014) are C++ programs focused on simulating sequence data for large pedigrees, addressing shared environments and correlated traits, such as blood pressure and BMI, in both case-control and quantitative trait designs.

Other simulation tools, such as simuRare (Xu *et al.* 2013)—an R package focused on rare variant analysis—and GPOPSIM (Zhang *et al.* 2015), which employs forward simulation under a mutation-drift equilibrium model, offer diverse functionalities. GPOPSIM supports the simulation of multiple correlated genetic traits under various assumptions, such as different population sizes and breeding ratios.

Finally, GCTA-Simu (Yang *et al.* 2011), part of the widely-used GCTA software, simulates GWAS data based on actual genotype information, allowing for arbitrary numbers of causal variants under additive quantitative or case-control liability threshold models.

A summary of the key features of these simulation tools is presented in Table 1.

**Table 1.** Overview of simulation software by software name and estimated year made available.^a^

Name	Year	C/C	Quant.	Resamp.	Recomb.	Adm.
HAP-SAMPLE2	2025	+	+	+	+	+
HAP-SAMPLE	2007	+	–	+	+	–
HAPSIMU	2008	+	+	–	+	+
HAPGEN2	2011	+	–	+	+	–
Hapnest	2023	+	+	+	+	–
simuGWAS	2011	+	–	–	+	+
GWAsimulator	2008	+	–	+	+	–
SEQSIMLA2	2013	+	+	–	+	–
simuRare	2013	+	–	+	+	–
GPOPSIM	2015	+	+	–	+	–
GCTA-Simu	2014	+	+	+	–	–

a “+” denotes presence of functionality; “–” denotes absence or unknown. The functionalities listed in the columns include support for simulating case/control traits, quantitative traits, resampling methods (e.g., bootstrapping from existing data), recombination, and admixture. Note that ‘Resampling’ refers to the software’s ability to perform resampling techniques on simulated or existing data, which may be relevant for generating null distributions or evaluating robustness.

**Figure 1.**
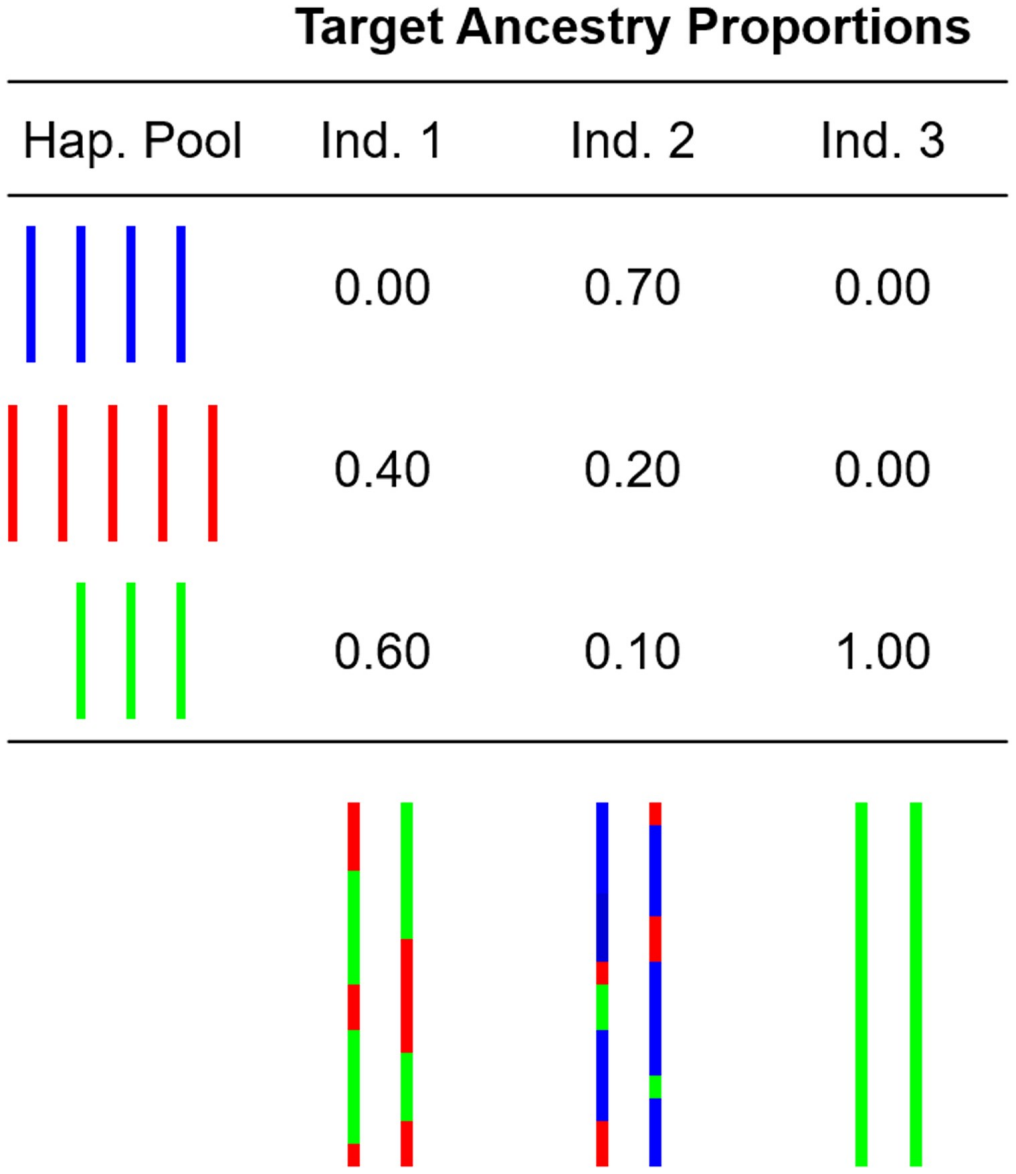
Example chromosomes for three individuals with admixture from three potential source populations. Individual 1 has two-way admixture between two source populations, Individual 2 has admixture from all three source populations, and Individual 3 has ancestry from one source population.

HAP-SAMPLE2 expands upon HAP-SAMPLE (Wright *et al.* 2007), a program designed to perform resampling of real autosomal chromosome data to produce simulated datasets for case-control studies. HAP-SAMPLE2 incorporates an admixture simulation process, enabling the generation of individuals with specified admixture proportions from different ancestral populations (Zhou *et al.* 2018). This enhances the realism of the simulated datasets, as portions of the simulated genomes are derived from distinct source populations according to user-defined admixture proportions. Figure 1 provides an example of the resulting chromosome compositions for individuals with varying degrees of admixture.

In addition to its ability to simulate common variants, HAP-SAMPLE2 includes functionality for performing burden tests on rare variants in both case-control and quantitative trait study designs. Burden testing aggregates minor allele counts across loci, which helps address challenges associated with low minor allele frequencies, often encountered with rare variants (Auer and Lettre 2015). While rare variants pose distinct challenges compared to common variants—such as reduced mutual correlation and the need for careful multiple testing corrections—burden testing offers a practical solution by assuming that rare variants tend to exert detrimental effects on fitness (Lee *et al.* 2014).

By integrating both common and rare variant simulation capabilities, HAP-SAMPLE2 provides a comprehensive platform for studying the genetic architecture of complex traits and diseases.

## 2 Methods

**HAP-SAMPLE2 offers four simulation types**: a two-by-two combination of common and rare variants, paired with either case/control or quantitative trait designs. While each of these simulation types has distinct features, they share core methodological and infrastructural aspects. The minimum recommended computing requirements for regional simulation are a computer with 16 Gb RAM and a 2.5 GHz processor.

In developing the software package, we used data from the 1000 Genomes Project (Auton et al. 2015). However, the methodology is flexible and can be adapted to other input datasets. The 1000 Genomes Project provides phased autosomal haplotype data in Variant Call Format (VCF) files for each chromosome, including 2,504 individuals.

In the simulations, we focus on biallelic loci, where “0” indicates the presence of the reference allele, and “1” denotes the alternative allele. By applying Hardy-Weinberg equilibrium assumptions, we can transition between allele frequencies and genotype frequencies. This assumption enables the storage of source and simulated haplotypes in a sparse vector format. As an optional pre-processing step, users can filter loci by minor allele frequency thresholds, retain every *n*th locus, or include specific loci based on reference SNP IDs.

Relative distances for loci in the 1000 Genomes dataset can be estimated through interpolation or extrapolation. These maps help identify recombination hotspots, where recombination is more likely to occur (Khil and Camerini-Otero 2010). We obtained genetic maps from the IMPUTE/IMPUTE2 website (Howie and Marchini 2015). For loci within the map range, linear interpolation was used, while for loci outside the range, extrapolation was performed via simple linear regression for each chromosome. Recombination probabilities for specific loci were calculated by converting centimorgan distances to probabilities (1 cM = 0.01 expected crossovers).

### 2.1 Case/control design for common variants

We begin by describing the case/control design for common variants, the primary simulation type in HAP-SAMPLE2. Since the four simulation types share core methodologies, we describe the case/control design in detail and then highlight differences between the other types.

In case/control designs, cases typically represent individuals with a disease, while controls do not. Let $\pi_{i}$ be the probability of disease for an individual *i*. A logistic regression model is used to express this as:

$$\ln(\frac{\pi_{i}}{1-\pi_{i}})=a_{i}^{T}\alpha_{s}+g_{is}\beta_{s},$$

where $a_{i}$ represents the admixture proportions of individual *i*, $\alpha_{s}$ is a vector of population-specific coefficients for locus *s*, $g_{is}$ is the genotype for locus *s* ($g_{is}\in 0,1,2$), and $\beta_{s}$ is the effect size of locus *s*. This can be reformulated as:

$$\pi_{i}=\frac{\;\text{exp}(a_{i}^{T}\alpha_{s}+g_{is}\beta_{s})}{1+\text{exp}(a_{i}^{T}\alpha_{s}+g_{is}\beta_{s})}=\frac{\;\text{exp}(z_{i})}{1+\text{exp}(z_{i})},$$

where $z_{i}$ represents the log-odds of disease.

In HAP-SAMPLE2, users specify case prevalence for each population, causal SNPs, logistic regression coefficients, and admixture proportions. The software calculates ancestry coefficients for each SNP and assigns genotypes for each simulated individual.

#### 2.1.1 Ancestry coefficients

The ancestry coefficients are solved using user-specified disease prevalence, allele frequencies, and genotype effect sizes. Alternative allele frequencies for each population are derived from source haplotype data, and genotype frequencies are calculated via Hardy-Weinberg equilibrium (Wright *et al.* 2007). For a population *k* and SNP *s*, the genotype frequencies are:

$$P_{k}(g_{is}=0)={\left(1-f_{ks}\right)}^{2},$$

 

$$P_{k}(g_{is}=2)=f_{ks}^{2},$$

 

$$P_{k}(g_{is}=1)=1-P_{k}(g_{is}=0)-P_{k}(g_{is}=2).$$

The probability that an individual from this population with genotype $g_{is}$ is a case is:

$$\frac{\;\text{exp}(\alpha_{ks}+g_{is}\beta_{s})}{1+\text{exp}(\alpha_{ks}+g_{is}\beta_{s})},$$

where $\alpha_{ks}$ is the intercept. Given this, we can solve for $\alpha_{ks}$ by numerically optimizing:

$$p_{k}=\frac{1}{N_{k}}\sum_{1}^{N_{k}}\frac{\;\text{exp}(\alpha_{ks}+g_{is}\beta_{s})}{1+\text{exp}(\alpha_{ks}+g_{is}\beta_{s})},$$

where $N_{k}$ is the population size. The result provides the ancestry coefficients $\alpha_{s}$ for each SNP. These ancestry coefficients vary slightly by causal locus to accommodate the constraints imposed by marginal disease probabilities (Supplementary File, Section S1, available as supplementary data at *Bioinformatics* online).

#### 2.1.2 Absolute genotype specification

Once the ancestry coefficients are determined, the next step is to simulate genotypes given the disease status and ancestry proportions. For each individual, the probability of disease given genotype and ancestry, $P(D_{i}=1|g_{is},a_{i})$, is calculated, and the probability of genotype given disease and ancestry, $P(g_{is}|D_{i}=1,a_{i})$, is derived through conditional probability calculations (Supplementary File, Section S1, available as supplementary data at *Bioinformatics* online).

#### 2.1.3 Haplotype construction

A Poisson approximation can be used to stochastically determine the number of crossovers for each haplotype for each individual. Let $L_{h}$ represent the number of loci on a given chromosome *h*, and $\theta_{hl}$ represent the recombination probability between locus *l* and l+1 on that chromosome. Then for each individual’s haplotype of chromosome *h*, the number of assigned crossovers for that chromosome, $r_{h}$, could be determined via a random draw from the Poisson distribution with Poisson parameter $\lambda_{h}=\sum_{l=1}^{L_{h}-1}\theta_{hl}$.

Furthermore, we introduce a floor of 1 such that each simulated chromosome experiences at least one crossover, and adjust the Poisson parameter accordingly to leave the mean number of crossovers unchanged. Although this requirement does not have a large impact on any downstream simulation results, this more closely matches the expectation that there occurs at least one crossover per human chromosome (Wilson and Hunt 2002). For each $\lambda_{h}$, we solve for a $\lambda_{h}^{*}$ such that $E\left[r_{h}\right]=1+E\left[r_{h}^{*}\right]$, where each $r_{h}^{*}$ is also Poisson distributed with Poisson parameter $\lambda^{*}$. The number of crossovers for a given individual on a given chromosome, $r_{h}^{*}$, can then be determined via a random draw from this adjusted distribution.

Table S1, Figs S2–S6, and Section S2.1 of the Supplementary File, available as supplementary data at *Bioinformatics* online illustrate the crossover simulations, the achievement of target ancestry proportions and admixture, and type I/power results for the case/control scenario.

### 2.2 Quantitative phenotype for common variants

Unlike for the case/control design, we are not conditioning upon the quantitative phenotype in drawing genotypes. Suppose an individual *i* has a phenotype value of $z_{i}$ for a given trait. Assuming normality of the trait and equal variance across sub-populations, we can write:

$$z_{i}∼\phi (a_{i}^{T}\alpha +g_{i}^{T}\beta ,\sigma^{2}),$$

where ϕ is the normal probability density function, $a_{i}$ and $g_{i}$ are the individual’s ancestry proportions and genotype, respectively, α contains ancestry-specific intercepts, β contains the user-specified loci effect sizes, and $\sigma^{2}$ the user-specified variance. In contrast to the case/control design, the quantitative phenotype simulation allows for multiple loci per chromosome to be designated as causal SNPs. Analogous to specifying disease prevalences in the case/control design, users can provide source population means for the quantitative trait, which are then used to solve for the ancestry intercepts $\alpha_{k}$ for each population.

#### 2.2.1 Ancestry coefficients for quantitative phenotypes

The ancestry coefficients are solved in a manner similar to the case/control design. However, instead of calculating coefficients for each SNP, we compute a single ancestry intercept $\alpha_{k}$ for each ancestral population *k*.

Let us assume there are *S* causal SNPs and thus $3^{S}=M$ combinations of genotypes. Let $g_{m},m\in \{1,\ldots ,M\}$, be a particular combination of genotypes, such that $g_{1}=\{0,\ldots ,0\}$ and $g_{M}=\{2,\ldots ,2\}$.

If $\mu_{k}$ is the user-specified mean trait value for population *k*, $f_{ks}$ is the alternative allele frequency for SNP *s* for population *k*, $P_{k}(g_{\mathrm{im}})$ is the genotype frequency for genotype $g_{m}$ among population *k*, and β is a vector of effect sizes for the causal SNPs, then we can express the phenotype for poluation *k* as:

$$z_{km}=\alpha_{k}+g_{m}\beta ,$$

 

$$\mu_{k}=\sum_{m=1}^{3^{S}}z_{km}P_{k}(g_{\mathrm{im}}).$$

Since $\alpha_{k}$ is the only unknown, we can solve for each $\alpha_{k}$ via numerical optimization. Once we have the ancestry intercepts α, we can use them to simulate phenotypes for individuals based on their genotypes and ancestry proportions (Section S2.2 of the Supplementary File, available as supplementary data at *Bioinformatics* online).

#### 2.2.2 Haplotype construction

Haplotype construction for the quantitative phenotype simulation is similar to the approach used in the case/control design. However, for quantitative traits, we no longer need to account for specific causal SNPs during the haplotype construction phase, as the phenotype values for each individual are computed after the genotypes are generated. Thus, the process involves constructing haplotypes and assigning genotypes without conditioning on phenotype values.

### 2.3 Case/control design for rare variants

In a rare variant case/control design, users specify ancestral population-specific case prevalences, similar to the common variants setup. Additionally, users define the chromosomes, mean effect size of a rare variant allele, weighting scheme, and minor allele threshold. Unlike the common variants design, there is no specification of effect sizes for specific loci.

HAP-SAMPLE2 supports two weighting schemes: Fixed and Madsen-Browning. For the Fixed scheme, rare allele counts are weighted uniformly:

$$w_{s}=\{\begin{array}{cc} 1 & \text{if}\;f_{s}<f_{\mathrm{thresh}}\\ 0 & \text{if}\;f_{s}\geq f_{\mathrm{thresh}} \end{array}.$$

In the Madsen-Browning scheme, weights are calculated as:

$$w_{s}=\frac{1}{{\left[\frac{f_{s}}{1-f_{s}}\right]}^{\frac{1}{2}}},$$

where $f_{s}$ is the minor allele frequency at locus *s* (Madsen and Browning 2009, Lee *et al.* 2014). The weights are adjusted so that the mean adjusted weight matches the user specification:

$$\beta_{s}=\beta_{*}\frac{Sw_{s}}{\sum_{s=1}^{S}w_{s}}.$$

Minor allele frequencies are averaged across population-specific frequencies, considering different evolutionary histories and mutational loads (Henn *et al.* 2015).

For each locus *s*, the burden score for individual *i* is:

$$c_{i}=\sum_{s=1}^{S}g_{is}\beta_{s}=g_{i}^{T}\beta ,$$

where $g_{is}$ is the genotype value (0, 1, or 2) for individual *i* at locus *s*, and $\beta_{s}$ is the adjusted weight.

#### 2.3.1 Ancestry coefficients

Ancestry-specific coefficients $\alpha_{k}$ are calculated for each population. For individual *i*, the probability $\pi_{i}$ of being a case is given by:

$$\ln\left(\frac{\pi_{i}}{1-\pi_{i}}\right)=\alpha_{k}+c_{i},$$

where $\alpha_{k}$ is solved to ensure that the mean probability for population *k* matches the specified prevalence $p_{k}$:

$$p_{k}=\frac{1}{N_{k}}\sum_{i=1}^{N_{k}}\frac{\;\text{exp}(\alpha_{k}+c_{i})}{1+\text{exp}(\alpha_{k}+c_{i})},$$

where $N_{k}$ is the number of individuals in population *k*. Optimization of $\alpha_{k}$ can be performed using R’s optimize.

#### 2.3.2 Haplotype construction

Once the genotype probabilities are computed, we proceed with haplotype construction for rare variants. The haplotypes are generated independently of disease status, using the population-specific allele frequencies for the rare variants. We construct haplotypes by sampling from these frequencies and then combine haplotypes to form genotypes for each individual.

After generating the haplotypes, we assign genotypes and subsequently adjust the disease status. The rare variants can thus exert a strong influence on the disease status, especially when the effect sizes are large.

### 2.4 Quantitative phenotype for rare variants

In the quantitative phenotype approach for rare variants, we first construct haplotypes and then determine the phenotypes of simulated individuals. Given the rare variant scores $c_{i}$ for each individual and intercept adjustments for each ancestral sub-population α, we can assign phenotype scores in a manner similar to common variants.

The phenotype for individual *i* is drawn from a normal distribution:

$$z_{i}∼\phi (a_{i}^{T}\alpha +c_{i},\sigma^{2}),$$

where α represents the ancestry coefficients, and $\sigma^{2}$ is the variance of the phenotype distribution.

To determine α, we use the user-specified means for the targeted ancestral populations, μ:

$$\mu_{k}=\frac{1}{N_{k}}\sum_{i=1}^{N_{k}}\left(\alpha_{k}+c_{i}\right),$$

where $N_{k}$ is the number of individuals in population *k*.

Thus, the phenotype score $z_{i}$ for each individual after haplotype construction is:

$$z_{i}=a_{i}^{T}\alpha +c_{i}+\epsilon_{i},$$

where $\epsilon_{i}$ is an independent draw from a normal distribution with mean 0 and variance $\sigma^{2}$. The standard deviation σ is specified by the user.

## 3 Conclusion

HAP-SAMPLE2 is a robust tool for simulating genotype-phenotype datasets, adept at handling both case-control studies and quantitative phenotypes, and particularly well-suited for large-scale datasets like those from the 1000 Genomes Project. Its capability to simulate admixed populations and introduce novel variation through artificial recombination ensures a realistic representation of complex population structures. The tool’s comprehensive documentation and vignette instructions enable researchers to seamlessly integrate HAP-SAMPLE2 into their workflows, regardless of their computational expertise. Additionally, HAP-SAMPLE2 supports burden testing for rare variants using fixed and Madsen-Browning weighting schemes, making it a valuable asset for rare variant association studies.

## Author Contributions

George Sun (Software, Writing—original draft, Writing—reviewing & editing), Bryan W. Ting (Methodology), Fred A. Wright (Funding acquisition, Writing—reviewing & editing), and Yi-Hui Zhou (Conceptualization, Methodology, Supervision, Writing—reviewing & editing, Funding acquisition)

## Supplementary Material

btaf333_Supplementary_Data

## Data Availability

Code and data are available at: https://github.com/M3dical/HAPSAMPLE2
