# Supplementary material for: HAP-SAMPLE2: data-based resampling for association studies with admixture
Source: Bioinformatics. 2025 Jun 13;41(6):btaf333. doi: 10.1093/bioinformatics/btaf333 (PMC12208071; doi:10.1093/bioinformatics/btaf333)
Supplement: btaf333_Supplementary_Data [file btaf333_supplementary_data.zip › vignette.pdf]

HAP-SAMPLE2

Dr. Yi-Hui Zhou's Group 2025-04-04

Load required packages and input functions. Specify the number of cores for parallel execution of R code.

```
if(!require("parallel")) install.packages("parallel")
if(!require("vcfR")) install.packages("vcfR")
if(!require("Matrix")) install.packages("Matrix")
if(!require("slam")) install.packages("slam")
if(!require("matrixStats")) install.packages("matrixStats")
if(!require("doParallel")) install.packages("doParallel")
if(!require("foreach")) install.packages("foreach")
if(!require("DirichletReg")) install.packages("DirichletReg")
if(!require("LEA")) install.packages("LEA")
if(!require("ggplot2")) install.packages("ggplot2")
registerDoParallel(cores=8)
source("D:/HAP-SAMPLE2/HAP-SAMPLE2_functions.R")
```

Example Simulations

Example simulations were performed using a thinned version of SNPs and individuals from 1000 Genomes. Phased haplotype data and information file with population codes were downloaded from 1000 Genomes website (https://ftp.1000genomes.ebi.ac.uk/vol1/ftp/release/20130502). Genetic maps were downloaded from the IMPUTE website (https://mathgen.stats.ox.ac.uk/impute/1000GP_Phase3.html). Process the VCF files and output SNP positions of alternative alleles for each individual haplotype, minor allele counts by individual haplotype, minor allele frequencies by population code, and SNP locations.

```
indir <- "D:/HAP-SAMPLE2/"
dir.create(file.path(indir,"processed"))
outdir <- "D:/HAP-SAMPLE2/processed/"
sample_filename <- "integrated_call_samples_v3.20130502.CEU_CHB_YRI.panel"
chr_vec <- 1:22
n_cores <- 8
for (i in chr_vec) {
  infilename <- paste0("1000G.chr",i,".recode.vcf.gz")
  vcfFile_to_rds(indir, outdir, infilename, max_ram=8, max_inds=310, sample_filename,
                 print_ind_count=TRUE, n_cores=n_cores, n_fixed_cols=9)
}
file.copy(from=paste0(indir,sample_filename), to=outdir)
writeAlleleFreqs_W(dir=outdir, chr_vec, sample_filename, n_cores=n_cores)
dir.create(file.path(indir,"sampleChromo"))
outdir <- "D:/HAP-SAMPLE2/sampleChromo/"
for (i in chr_vec) { file.copy(from=paste0(indir,"genetic_map_chr",i,"_combined_b37.txt"),
                               to=outdir) }
file.copy(from=paste0(indir,sample_filename), to=outdir)
```

Case/Control Design for Common Variants

1,000 cases and 1,000 controls were simulated. For cases, 300 individuals were designated to have target admixture proportions of 100%, 100 each for CEU, CHB, and YRI. 300 individuals were targeted to have 50% admixture proportions, 100 for each pairwise combination between CEU, CHB, and YRI. 100 individuals were designated to have 1/3 admixture between the three. 300 individuals had target admixture proportions generated from the Dirichlet distribution with means of 1/3. Target admixture proportions for the controls were specified the same way.

```
elig_pops <- c("CEU","CHB","YRI")
set.seed(1000)
adm_mat <- matrix(0,nrow=1000,ncol=length(elig_pops))
colnames(adm_mat) <- elig_pops
adm_mat[1:100, "CEU"] <- 1
adm_mat[101:200, "CHB"] <- 1
adm_mat[201:300, "YRI"] <- 1
adm_mat[301:400, c("CEU","CHB")] <- 0.5
adm_mat[401:500, c("CEU","YRI")] <- 0.5
adm_mat[501:600, c("CHB","YRI")] <- 0.5
adm_mat[601:700, c("CHB","CEU","YRI")] <- 1/3
adm_mat[701:1000, c("CHB","CEU","YRI")] <- rdirichlet(300, alpha=c(1/3,1/3,1/3))
group <- c(rep(1, 100), rep(2, 100), rep(3, 100), rep(4, 100),
           rep(5, 100), rep(6, 100), rep(7, 100), rep(8, 300))
desc <- c("CEU", "CHB", "YRI", "CEU/CHB", "CEU/YRI", "CHB/YRI", "CEU/CHB/YRI",
          "Dirichlet")
target <- data.frame(Group=1:8, Type=c(rep("Fixed",7),"Dirichlet"),
                     Inds.=c(rep(100,7),300),
                     CEU=c("1","0","0","1/2","1/2","0","1/3","1/3"),
                     CHB=c("0","1","0","1/2","0","1/2","1/3","1/3"),
                     YRI=c("0","0","1","0","1/2","1/2","1/3","1/3"))
print(target)
```

```
##   Group      Type Inds. CEU CHB YRI
## 1     1     Fixed   100   1   0   0
## 2     2     Fixed   100   0   1   0
## 3     3     Fixed   100   0   0   1
## 4     4     Fixed   100 1/2 1/2   0
## 5     5     Fixed   100 1/2   0 1/2
## 6     6     Fixed   100   0 1/2 1/2
## 7     7     Fixed   100 1/3 1/3 1/3
## 8     8 Dirichlet   300 1/3 1/3 1/3
```

Genetic Map for Recombination Probabilities

Specify 22 causal SNPs (SNPs associated with trait values), one per chromosome. Retain these SNPs, and filter SNPs with a minor allele threshold >0.02 in 1000 Genomes for simulation.

```
retain_snp <- c("rs72638544","rs7579608","rs10510310","rs7685911","rs1901498","rs4960131",
                "rs10237521","rs4433167","rs7865441","rs4584478","rs9804657","rs55768228",
                "rs7991629","rs28721413","rs7180505","rs9935770","rs11247560","rs62078854",
                "rs12980335","rs156338","rs9980561","rs5746362")
indir <- "D:/HAP-SAMPLE2/processed/"
```

```
sampleChromo_W(chr_vec, indir, outdir, sample_filename, retain_every=1, retain_snp=retain_snp,
               maf_threshold=0.02, n_cores=n_cores)
```

Starting with genetic maps containing SNP base positions and corresponding relative distances in centimorgans (cM), estimate relative distances for your set of SNPs via interpolation and/or extrapolation. For SNPs within the range of the genetic map, linear interpolation was performed using contiguous points from the map. For SNPs outside the range, extrapolation was done using a simple linear regression informed by all of the SNPs in the genetic map, performed for each chromosome. Then obtain SNP recombination probabilities by taking the difference between relative distances and converting the centimorgans to probabilities.

```
dir <- "D:/HAP-SAMPLE2/sampleChromo/"
loci_info_list <- makeLociInfo_W(chr_vec, dir)
loci_info <- loci_info_list$loci_info
chr_recomb <- loci_info_list$chr_recomb
```

Calculate allele and genotype frequencies of the causal SNPs by population group for CEU, CHB, and YRI individuals in 1000 Genomes.

```
ChrAlleleFreqs <- calcChrAlleleFreqs_W(retain_snp, loci_info, chr_vec, dir, sample_filename,
                                       n_cores=n_cores, elig_pops)
sample_ma_list <- ChrAlleleFreqs$sample_ma_list
snp_map <- ChrAlleleFreqs$snp_map
AlleleFreq_by_pop <- calcAlleleFreq_by_pop(sample_ma_list, snp_map)
gf_by_anc <- makeGenFreqAnc(ma_freq=AlleleFreq_by_pop)
colnames(gf_by_anc) <- elig_pops
```

Ancestry Coefficients

Specify a disease prevalence of 0.2, 0.1, and 0.3 for CEU, CHB, and YRI, respectively. Specify effect sizes of [1,0,-1,0] for the first 20 chromosomes, and [0,0] for the last two chromosomes. Given disease prevalence, alternative allele frequency, and the genotype coefficient (effect size), solve for the ancestry coefficients in a logistic regression model for each causal SNP.

```
spec_prev <- c(0.2,0.1,0.3)
names(spec_prev) <- elig_pops
B1 <- c(rep(c(1,0,-1,0),5),0,0)
names(B1) <- retain_snp
B0Est_case <- solveB0Est_W(mu_vec=spec_prev, gf_by_anc, B1, type="bin", lower=-8, upper=8)
B0Est_cont <- -B0Est_case
```

Absolute Genotypes in Cases and Controls

We wish to have an absolute genotype specification, the probability of genotype given disease and ancestry, as simulated individuals have a pre-specified disease status and user-provided ancestry proportions. Given the ancestry vector solutions for each combination of ancestral population and SNP, convert the inputs into the absolute genotype specification via typical conditional probability calculations. Since we assume loci are independent across chromosomes and limit each chromosome to one causal SNP, this formulation allows us to efficiently draw genotypes, i.e. determine whether an individual has a 0, 1, or 2 for each SNP.

```
case_genos <- drawGenotypes_W(B0Est=B0Est_case, B1=B1, spec_prev=spec_prev,
                              gf_by_anc, adm_mat)$drawnGenos
cont_genos <- drawGenotypes_W(B0Est=B0Est_cont, B1=-B1, spec_prev=1-spec_prev,
                              gf_by_anc, adm_mat)$drawnGenos
```

Output mean allele counts of the causal SNPs for case and control individuals post-simulation. As expected, for SNPs with positive effect sizes, cases had noticeably higher mean counts than controls. For SNPs with negative effect sizes, controls analogously had higher mean counts than cases. When the effect sizes for SNPs were 0, mean counts were similar between cases and controls.

```
freq_table <- data.frame(chr_vec,retain_snp,B1,colMeans(case_genos),colMeans(cont_genos))
colnames(freq_table) <- c("Chr.","SNP","Effect Size","Cases","Controls")
print(freq_table)
```

```
##            Chr.        SNP Effect Size Cases Controls
## rs72638544    1 rs72638544           1 0.241    0.090
## rs7579608     2  rs7579608           0 0.218    0.209
## rs10510310    3 rs10510310          -1 0.086    0.244
## rs7685911     4  rs7685911           0 0.360    0.326
## rs1901498     5  rs1901498           1 1.376    0.924
## rs4960131     6  rs4960131           0 0.922    0.917
## rs10237521    7 rs10237521          -1 0.453    0.841
## rs4433167     8  rs4433167           0 0.707    0.714
## rs7865441     9  rs7865441           1 1.208    0.744
## rs4584478    10  rs4584478           0 0.543    0.517
## rs9804657    11  rs9804657          -1 0.487    0.941
## rs55768228   12 rs55768228           0 0.550    0.565
## rs7991629    13  rs7991629           1 0.562    0.270
## rs28721413   14 rs28721413           0 0.736    0.782
## rs7180505    15  rs7180505          -1 0.330    0.654
## rs9935770    16  rs9935770           0 0.799    0.835
## rs11247560   17 rs11247560           1 1.404    0.909
## rs62078854   18 rs62078854           0 0.492    0.507
## rs12980335   19 rs12980335          -1 0.776    1.224
## rs156338     20   rs156338           0 0.794    0.713
## rs9980561    21  rs9980561           0 0.776    0.766
## rs5746362    22  rs5746362           0 0.656    0.644
```

Haplotype Construction

Starting with the absolute genotypes of the causal SNPs and the filtered SNPs in 1000 Genomes, perform haplotype resampling to simulate individuals with user-specified ancestral population proportions, resulting in realistic linkage disequilibrium patterns and allele frequencies. A Poisson approximation can be used to stochastically determine the number of crossovers for each haplotype for each individual, with a floor of 1 such that each simulated chromosome experiences at least one crossover.

```
dis_chr <- rep("yes",length(chr_vec))
case_cGM <- createGenoMult(dis_chr, dir, loci_info, sample_ma_list, adm_mat,
                           start_genos=case_genos, snp_map, chr_recomb, prefix="case",
                           write_genos=T, return_genos=F, write_every=1, n_cores=n_cores, compress=F)
cont_cGM <- createGenoMult(dis_chr, dir, loci_info, sample_ma_list, adm_mat,
```

```
                                start_genos=cont_genos, snp_map, chr_recomb, prefix="cont",
                                write_genos=T, return_genos=F, write_every=1, n_cores=n_cores, compress=F)
```

Estimate Admixture Proportions

Randomly sample 20% of simulated SNPs from each of the 22 chromosomes. Calculate the estimated admixture proportions for cases using the snmf function in the LEA R package. The admixture calculations were done in unsupervised fashion, i.e. no reference populations were designated as the three ancestral populations. The mean estimated admixture proportions by group indeed reflected the target admixture proportions. Within each group, sort individuals by estimated admixture proportions. Generate an admixture plot.

```
set.seed(1000)
case_geno <- NULL
cont_geno <- NULL
for (c in chr_vec) {
  case_infiles <- case_cGM$fileList[[c]]
  n_files <- length(case_infiles)
  case_inhap <- readRDS(paste0(dir,case_infiles[1]))
  snps <- sample(1:length(case_inhap),round(length(case_inhap)*0.20))
  snps <- sort(snps)
  case_counts <- case_inhap[snps]
  cont_infiles <- cont_cGM$fileList[[c]]
  cont_inhap <- readRDS(paste0(dir,cont_infiles[1]))
  cont_counts <- cont_inhap[snps]
  for (i in 2:n_files) {
    case_inhap <- readRDS(paste0(dir,case_infiles[i]))
    case_counts <- rbind(case_counts,case_inhap[snps])
    cont_inhap <- readRDS(paste0(dir,cont_infiles[i]))
    cont_counts <- rbind(cont_counts,cont_inhap[snps])
  }
  case_geno <- cbind(case_geno,case_counts)
  cont_geno <- cbind(cont_geno,cont_counts)
}
indir <- "D:/HAP-SAMPLE2/"
write.geno(case_geno,paste0(indir,"case.geno"))
```

```
case_snmf <- snmf(paste0(indir,"case.geno"), project="new", K=3, seed=1000)
```

```
Q <- Q(case_snmf, K=3)
Q <- as.data.frame(Q)
colnames(Q) <- elig_pops
Q.1.100 <- Q[1:100,]
Q.101.200 <- Q[101:200,]
Q.201.300 <- Q[201:300,]
Q.301.400 <- Q[301:400,]
Q.401.500 <- Q[401:500,]
Q.501.600 <- Q[501:600,]
Q.601.700 <- Q[601:700,]
Q.701.1000 <- Q[701:1000,]
Q.1.100 <- Q.1.100[order(Q.1.100$CEU,decreasing=T),]
Q.101.200 <- Q.101.200[order(Q.101.200$CHB,decreasing=T),]
```

```
Q.201.300 <- Q.201.300[order(Q.201.300$YRI,decreasing=T),]
Q.301.400 <- Q.301.400[order(Q.301.400$CEU,Q.301.400$CHB,decreasing=T),]
Q.401.500 <- Q.401.500[order(Q.401.500$CEU,Q.401.500$YRI,decreasing=T),]
Q.501.600 <- Q.501.600[order(Q.501.600$CHB,Q.501.600$YRI,decreasing=T),]
Q.601.700 <- Q.601.700[order(Q.601.700$CEU,Q.601.700$CHB,Q.601.700$YRI,decreasing=T),]
Q.701.1000 <- Q.701.1000[order(Q.701.1000$CEU,Q.701.1000$CHB,Q.701.1000$YRI,decreasing=T),]
Q <- rbind(Q.1.100,Q.101.200,Q.201.300,Q.301.400,Q.401.500,Q.501.600,Q.601.700,Q.701.1000)
Q <- data.frame(ind=rep(1:1000,3),pop=c(rep("CEU",1000),rep("CHB",1000),rep("YRI",1000)),
                prop=c(Q$CEU,Q$CHB,Q$YRI))
```

```
ggplot(Q,aes(x=ind,y=prop,fill=pop)) +
  geom_bar(position="fill",stat="identity") +
  scale_fill_manual(values=c("red","green","blue"),name=NULL) +
  scale_x_continuous(breaks=c(0,300,600,700,1000),labels=c("0","300","600","700","1000")) +
  scale_y_continuous(breaks=c(0,0.2,0.4,0.6,0.8,1.0),labels=c("0","0.2","0.4","0.6","0.8","1.0")) +
  xlab("One-Way                    Two-Way                                Three-Way             ") +
  ylab("Admixture Proportion") +
  theme_classic()
```

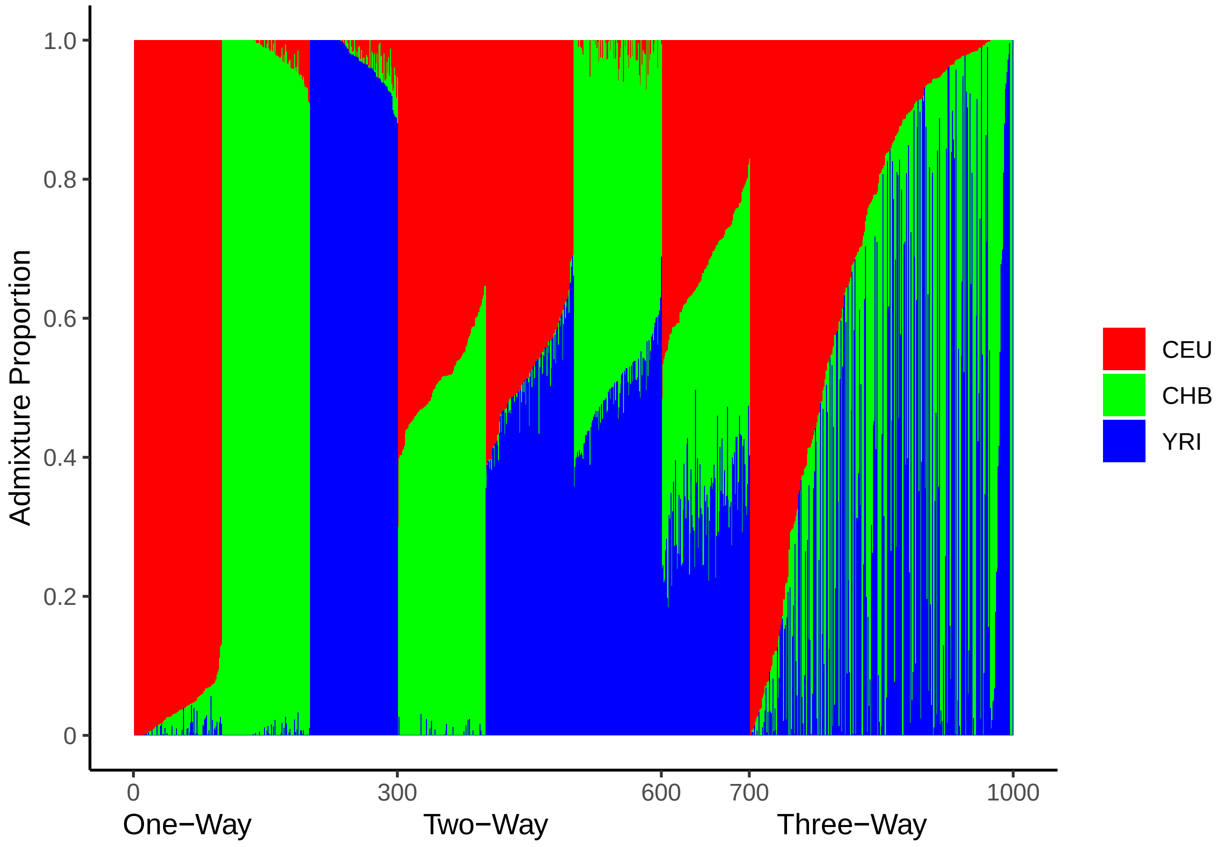

PCA using the same random sample of SNPs in cases, as another way to look at admixture results across groups, and plot the first two PCs. Individuals with targeted proportions of 100% of the ancestral populations indeed tightly cluster among themselves and comprise the nodes of a triangle, and individuals with 50% of two populations form the edges. Individuals with targeted proportions of 1/3 each are found in the center of the triangle, and the Dirichlet-targeted individuals are scattered across the triangle.

```r
pca <- prcomp(case_geno)
PC1.prop <- 100*signif(summary(pca)$importance["Proportion of Variance","PC1"],digits=2)
PC2.prop <- 100*signif(summary(pca)$importance["Proportion of Variance","PC2"],digits=2)
pca <- as.data.frame(pca$x)
pca <- pca[,1:2]
pca$group <- c(rep("CEU",100),rep("CHB",100),rep("YRI",100),rep("CEU/CHB",100),rep("CEU/YRI",100),
               rep("CHB/YRI",100),rep("CEU/CHB/YRI",100),rep("Dirichlet",300))
pca$group <- factor(pca$group, levels=c("CEU","CHB","YRI","CEU/CHB","CEU/YRI",
                                        "CHB/YRI","CEU/CHB/YRI","Dirichlet"))
ggplot(pca,aes(x=PC1,y=PC2,color=group)) +
  geom_point() +
  scale_color_manual(values=c("red","yellow","blue","orange","purple","green","lightgrey","darkgrey"),
                     name=NULL) +
  xlab(paste0("PC1 (",PC1.prop,"%)")) +
  ylab(paste0("PC2 (",PC2.prop,"%)")) +
  theme_classic()
```

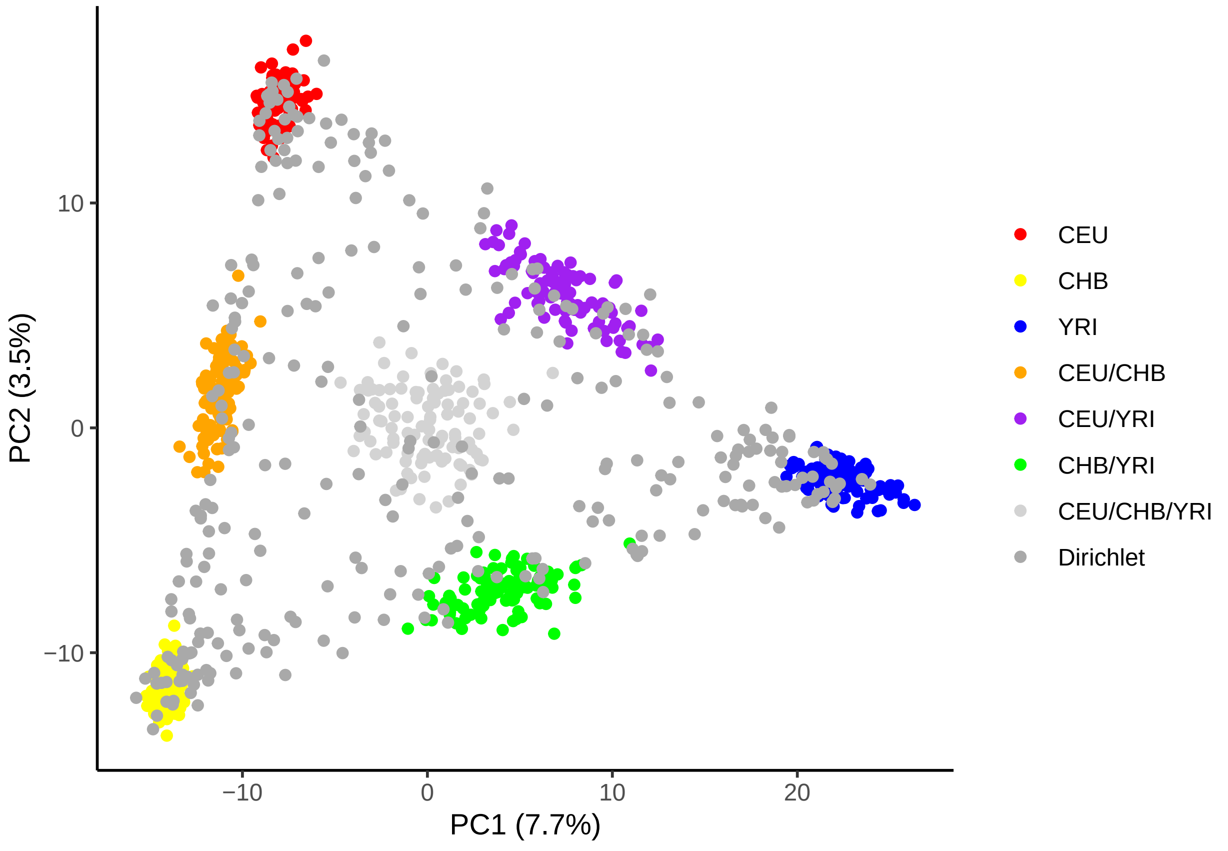

The 300 case individuals with Dirichlet-generated admixture proportions are depicted in scatterplots. Each plot compares targeted versus realized admixture for a given ancestral source population. Targeted proportions were those drawn from the Dirichlet distribution, and realized admixture proportions were provided by sNMF.

```r
targeted <- as.data.frame(adm_mat[701:1000,])
realized <- Q(case_snmf, K=3)
realized <- as.data.frame(realized)
realized <- realized[701:1000,]
```

```
colnames(realized) <- elig_pops
CEU.Rsq <- 100*signif(cor(targeted$CEU,realized$CEU)^2,digits=3)
CHB.Rsq <- 100*signif(cor(targeted$CHB,realized$CHB)^2,digits=3)
YRI.Rsq <- 100*signif(cor(targeted$YRI,realized$YRI)^2,digits=3)
par(mfrow=c(1,3))
plot(targeted$CEU,realized$CEU,xlab="Targeted Prop. CEU",ylab="Realized Prop. CEU",pch=19)
text(x=0.1,y=1,paste0("R-Sq=",CEU.Rsq,"%"))
plot(targeted$CHB,realized$CHB,xlab="Targeted Prop. CHB",ylab="Realized Prop. CHB",pch=19)
text(x=0.1,y=1,paste0("R-Sq=",CHB.Rsq,"%"))
plot(targeted$YRI,realized$YRI,xlab="Targeted Prop. YRI",ylab="Realized Prop. YRI",pch=19)
text(x=0.1,y=1,paste0("R-Sq=",YRI.Rsq,"%"))
```

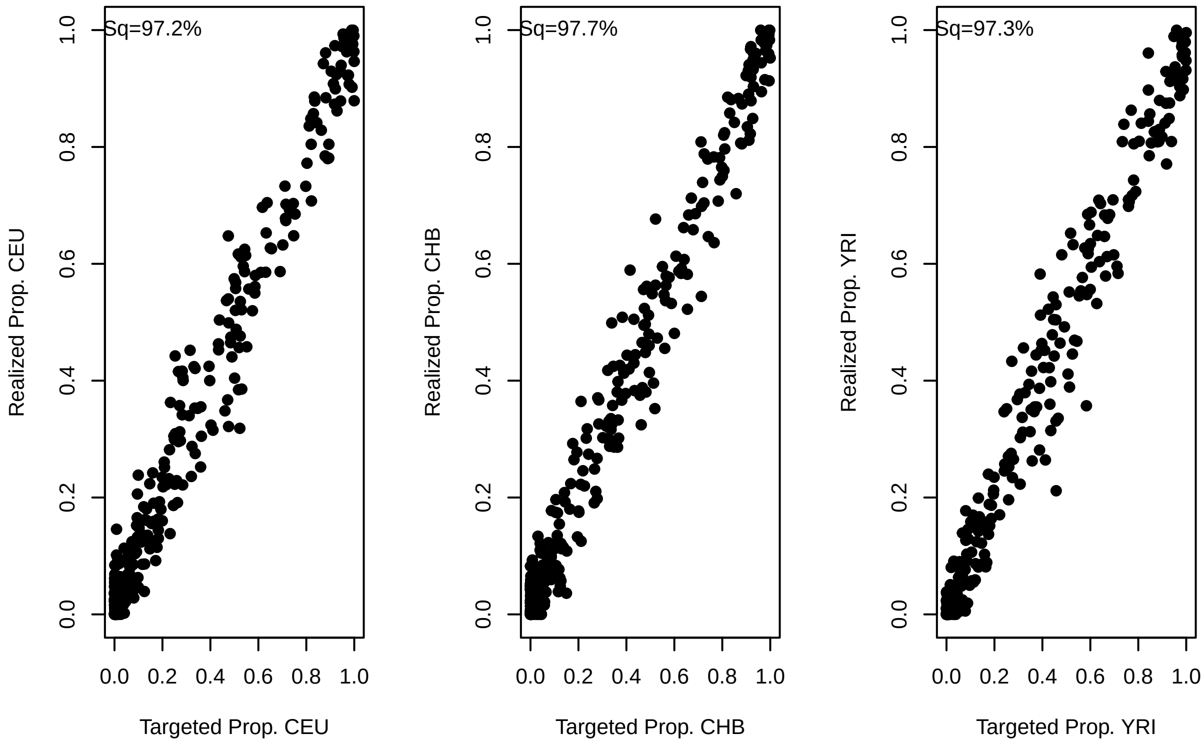

GWAS Analysis of Case/Control Phenotype

2,500 SNPs were randomly chosen to compute the first four principal components and were used as covariates. The 1,000 closest loci to each causal SNP were used in the GWAS to form windows. GWAS was conducted upon the simulated genotypes and phenotypes (a binary variable with 1 for cases, 0 for controls).

```
set.seed(1000)
geno <- rbind(case_geno,cont_geno)
geno <- geno[,sample(1:ncol(geno),2500)]
pca <- prcomp(geno)
pca <- as.data.frame(pca$x)
pca <- pca[,1:4]
```

```r
snpWindow <- snpWindows_W(chr_vec, retain_snp, loci_info, windowSize=1000)
case_gwas <- NULL
cont_gwas <- NULL
for (c in chr_vec) {
  case_infiles <- case_cGM$fileList[[c]]
  cont_infiles <- cont_cGM$fileList[[c]]
  n_files <- length(case_infiles)
  snps <- snpWindow[which(snpWindow$chr==c),]
  case_geno <- NULL
  cont_geno <- NULL
  for (i in 1:n_files) {
    case_inhap <- readRDS(paste0(dir,case_infiles[i]))
    case_geno <- rbind(case_geno,case_inhap[snps$locus_pos])
    cont_inhap <- readRDS(paste0(dir,cont_infiles[i]))
    cont_geno <- rbind(cont_geno,cont_inhap[snps$locus_pos])
  }
  colnames(case_geno) <- snps$name
  case_gwas <- cbind(case_gwas,case_geno)
  colnames(cont_geno) <- snps$name
  cont_gwas <- cbind(cont_gwas,cont_geno)
}
samples_gwas <- rbind(case_gwas,cont_gwas)
gwas_df <- data.frame(y=c(rep(1,1000),rep(0,1000)),pca,samples_gwas)
snpWindow$name <- gsub(";",".",snpWindow$name)
gwas_p <- gwas(gwas_df, y_name="y", cov_names=c("PC1","PC2","PC3","PC4"), s_names=snpWindow$name,
               type="logit", n_cores=n_cores)
```

Manhattan plot displays GWAS results. Causal SNP is highlighted in magenta. The 1,000 loci closest to the causal SNP are depicted in each window. Grey dashed line corresponds to a Bonferroni-corrected p-value of 0.05, applied at the genome-wide level of 84.4 million. R-squared values correspond to the degree of linkage disequilibrium for the loci with the nearest causal SNP. We see that in each window containing a causal SNP with an effect size of -1 or 1, the -log10(p-values) peak at that SNP. In contrast, no peaks are found in the windows where the causal SNPs had an effect size of 0. Loci closer to the causal SNPs had higher rates of LD, as conveyed via R-squared values.

```r
LD <- calcLD(center_locus=snpWindow$center_locus, quant_sub=t(samples_gwas), windows=snpWindow$window)
LD2 <- LD^2
LD2_spaced <- makeSpacedGroups(LD2, snpWindow$window, spaceSize=500)
gwas_p_spaced <- makeSpacedGroups(gwas_p, snpWindow$window, spaceSize=500)
col_spaced <- rep(NA,length(LD2_spaced))
names(col_spaced) <- names(LD2_spaced)
col_spaced[LD2_spaced>0.999] <- "magenta"
col_spaced[LD2_spaced>=0.8 & LD2_spaced<0.999] <- "red"
col_spaced[LD2_spaced>=0.6 & LD2_spaced<0.8] <- "orange"
col_spaced[LD2_spaced>=0.4 & LD2_spaced<0.6] <- "green"
col_spaced[LD2_spaced>=0.2 & LD2_spaced<0.4] <- "cyan"
col_spaced[LD2_spaced<0.2] <- "blue"
plot_gwas(p_values=gwas_p_spaced, denom=8.44E7, cols=col_spaced)
```

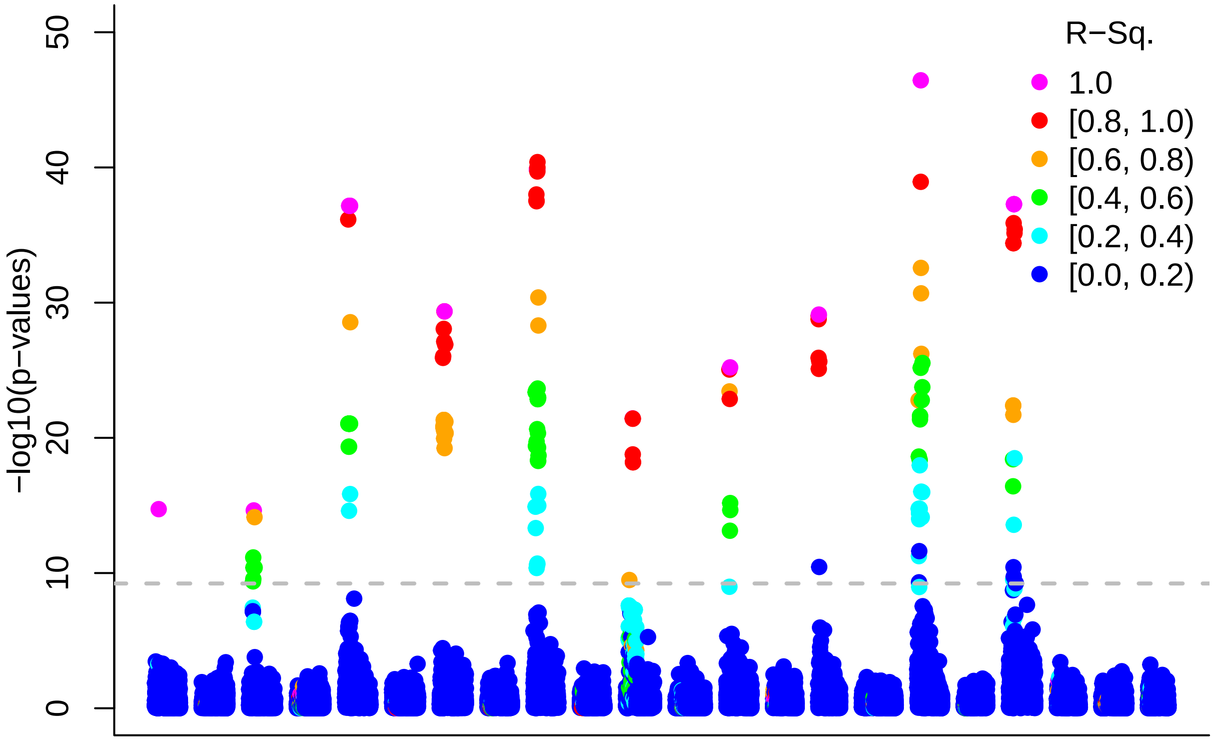

Quantitative Phenotype for Common Variants

Using a similar admixture set-up as for the case/control design, 2,000 individuals were simulated for a quantitative phenotype. Loci rs12980335, rs156338, rs9980561, rs6516850, rs2838791, and rs5746362 were specified as the causal loci, with effect sizes of 5, 0, 10, 0, 15, and 0 respectively. The specified population means for CEU, CHB, and YRI were 100, 105, and 85, respectively. The specified standard deviation was 15.

```
retain_snp <- c("rs12980335","rs156338","rs9980561","rs6516850","rs2838791","rs5746362")
chr_vec <- 19:22
loci_info <- loci_info[chr_vec]
chr_recomb <- chr_recomb[chr_vec]
ChrAlleleFreqs <- calcChrAlleleFreqs_W(retain_snp, loci_info, chr_vec, dir,
                                       sample_filename, n_cores=n_cores, elig_pops)
sample_ma_list <- ChrAlleleFreqs$sample_ma_list
snp_map <- ChrAlleleFreqs$snp_map
AlleleFreq_by_pop <- calcAlleleFreq_by_pop(sample_ma_list, snp_map)
gf_by_anc <- makeGenFreqAnc(ma_freq=AlleleFreq_by_pop)
colnames(gf_by_anc) <- elig_pops
```

```
mu_vec <- c(100,105,85)
names(mu_vec) <- elig_pops
sd <- 15
B1 <- c(5,0,10,0,15,0)
```

```r
names(B1) <- retain_snp
lower <- mu_vec - sd*5
upper <- mu_vec + sd*5
B0Est <- solveB0Est_both(mu_vec, gf_by_anc, B1, type="cont", lower, upper, max_combs=10^6)$B0Est
quant_adm_mat <- rbind(adm_mat,adm_mat)
quant_genos <- matrix(0, nrow=nrow(quant_adm_mat), ncol=length(retain_snp))
colnames(quant_genos) <- retain_snp
dis_chr <- rep("no",length(chr_vec))
quant_cGM <- createGenoMult(dis_chr, dir, loci_info, sample_ma_list, adm_mat=quant_adm_mat,
                            start_genos=quant_genos, snp_map, chr_recomb, prefix="quant",
                            write_genos=T, return_genos=F, write_every=1, n_cores=n_cores, compress=F)
snpWindow <- snpWindows_W(chr_vec, retain_snp, loci_info, windowSize=300)
gwasSampleList <- make_inSampleList(in_df=snpWindow)
quantOut <- subsettedGenoMat(dir, obj_cGM=quant_cGM, inSampleList=gwasSampleList)
quant_geno <- quantOut$genoMat
colnames(quant_geno) <- gsub(";",".",colnames(quant_geno))
```

1,000 SNPs were randomly chosen to compute the first four principal components. Manhattan plot displays GWAS results, conducted upon the simulated genotypes and phenotypes, using the first four principal components as covariates. As seen for the case/control design for common variants, the points in the windows peak where causal SNPs with non-zero effect sizes are found. Similarly, proximity of loci to the causal SNPs is positively associated with higher rates of LD.

```r
gwas_pca_sample <- sample.int(ncol(quant_geno), 1000)
gwas_pca <- prcomp(quant_geno[,gwas_pca_sample])
quantPheno <- quant_adm_mat[, elig_pops]%*%B0Est + quant_geno[, retain_snp]%*%B1 +
  rnorm(nrow(quant_adm_mat), 0, sd)
gwas_df <- data.frame(y=quantPheno,gwas_pca$x[,1:4],quant_geno)
gwas_p_pc <- gwas(gwas_df, y_name="y", cov_names=c("PC1","PC2","PC3","PC4"),
                  s_names=colnames(quant_geno), type="quant", n_cores=n_cores)
gwas_p_no_pc <- gwas(gwas_df, y_name="y", cov_names=NULL, s_names=colnames(quant_geno),
                     type="quant", n_cores=n_cores)
LD <- calcLD(center_locus=snpWindow$center_locus, quant_sub=t(quant_geno), windows=snpWindow$window)
LD2 <- LD^2
LD2_spaced <- makeSpacedGroups(LD2, snpWindow$window, spaceSize=100)
gwas_p_pc_spaced <- makeSpacedGroups(gwas_p_pc, snpWindow$window, spaceSize=100)
col_spaced <- rep(NA,length(LD2_spaced))
names(col_spaced) <- names(LD2_spaced)
col_spaced[LD2_spaced>0.999] <- "magenta"
col_spaced[LD2_spaced>=0.8 & LD2_spaced<0.999] <- "red"
col_spaced[LD2_spaced>=0.6 & LD2_spaced<0.8] <- "orange"
col_spaced[LD2_spaced>=0.4 & LD2_spaced<0.6] <- "green"
col_spaced[LD2_spaced>=0.2 & LD2_spaced<0.4] <- "cyan"
col_spaced[LD2_spaced<0.2] <- "blue"
plot_gwas(p_values=gwas_p_pc_spaced, denom=8.44E7, cols=col_spaced)
```

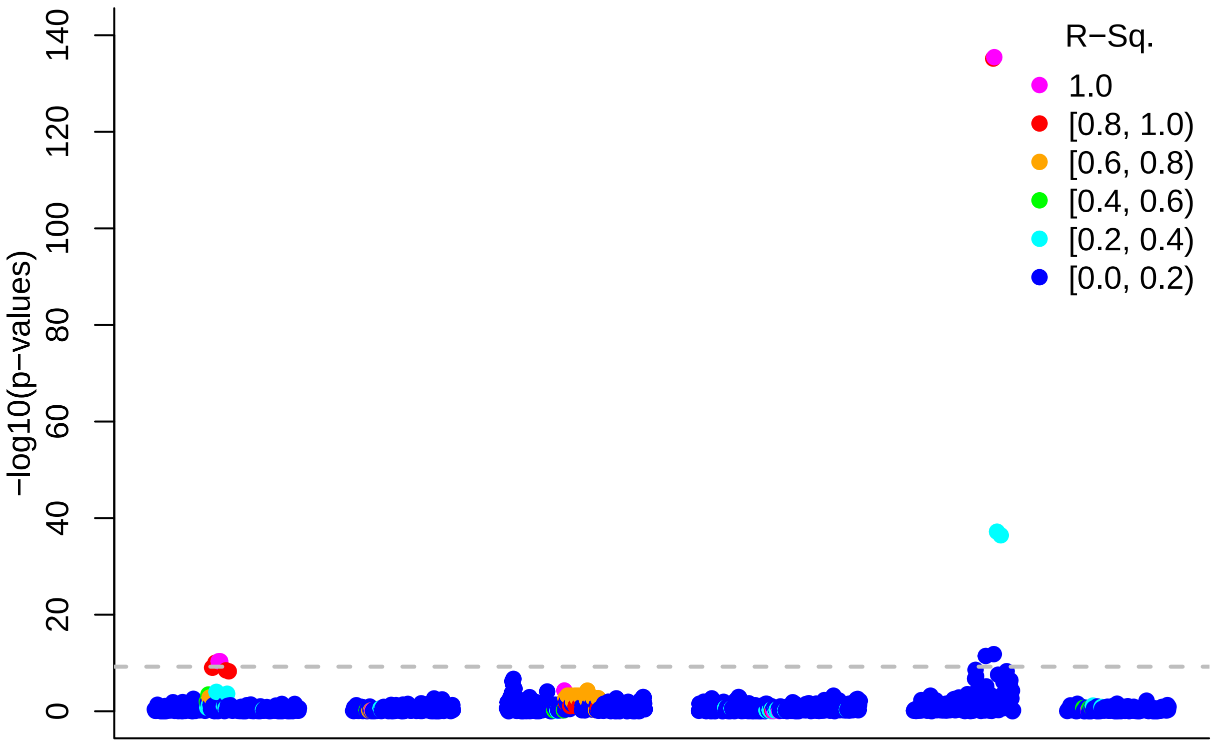

The simulation for a quantitative phenotype with common variants was re-run, this time with the effect sizes for the SNPs being all 0, and the other settings left unchanged. The q-q plot illustrates that neglecting to account for population stratification can result in more false positives, as the grey line (corresponding to -log10 p-values of the GWAS without the first four PCs as covariates) diverges from the unit line much sooner than does the blue line (corresponding to -log10 p-values of the GWAS including the first four PCs as covariates).

```
B1 <- c(0,0,0,0,0,0)
names(B1) <- retain_snp
lower <- mu_vec - sd*5
upper <- mu_vec + sd*5
B0Est <- solveB0Est_both(mu_vec, gf_by_anc, B1, type="cont", lower, upper, max_combs=10^6)$B0Est
quant_adm_mat <- rbind(adm_mat,adm_mat)
quant_genos <- matrix(0, nrow=nrow(quant_adm_mat), ncol=length(retain_snp))
colnames(quant_genos) <- retain_snp
dis_chr <- rep("no",length(chr_vec))
quant_cGM <- createGenoMult(dis_chr, dir, loci_info, sample_ma_list, adm_mat=quant_adm_mat,
                            start_genos=quant_genos, snp_map, chr_recomb, prefix="quant",
                            write_genos=T, return_genos=F, write_every=1,
                            n_cores=n_cores, compress=F)
snpWindow <- snpWindows_W(chr_vec, retain_snp, loci_info, windowSize=1000)
gwasSampleList <- make_inSampleList(in_df=snpWindow)
quantOut <- subsettedGenoMat(dir, obj_cGM=quant_cGM, inSampleList=gwasSampleList)
quant_geno <- quantOut$genoMat
colnames(quant_geno) <- gsub(";",".",colnames(quant_geno))
gwas_pca_sample <- sample.int(ncol(quant_geno), 1000)
```

```r
gwas_pca <- prcomp(quant_geno[,gwas_pca_sample])
quantPheno <- quant_adm_mat[, elig_pops]%*%B0Est + quant_geno[, retain_snp]%*%B1 +
  rnorm(nrow(quant_adm_mat), 0, sd)
gwas_df <- data.frame(y=quantPheno,gwas_pca$x[,1:4],quant_geno)
gwas_p_pc <- gwas(gwas_df, y_name="y", cov_names=c("PC1","PC2","PC3","PC4"),
                  s_names=colnames(quant_geno), type="quant", n_cores=n_cores)
gwas_p_no_pc <- gwas(gwas_df, y_name="y", cov_names=NULL, s_names=colnames(quant_geno),
                     type="quant", n_cores=n_cores)
p_pc_sort <- -log10(rev(sort(gwas_p_pc)))
p_no_pc_sort <- -log10(rev(sort(gwas_p_no_pc)))
expected_p <- rev(-log10((1:ncol(quant_geno))/ncol(quant_geno)))
gwas_qq <- data.frame(matrix(c(expected_p, expected_p, p_pc_sort, p_no_pc_sort), ncol = 2))
colnames(gwas_qq) <- c("expected", "observed")
gwas_qq_col_1 <- rep("blue", ncol(quant_geno))
gwas_qq_col_2 <- rep("grey", ncol(quant_geno))
gwas_qq[, "col"] <- c(gwas_qq_col_1, gwas_qq_col_2)
gwas_qq <- gwas_qq[order(gwas_qq[, "col"]),]
lim <- trunc(max(gwas_qq[, c("expected", "observed")])) + 1
par(mar=c(4,4,.5,1), ps=12)
plot(x = gwas_qq[,"expected"], y = gwas_qq[,"observed"], col = gwas_qq[, "col"], cex = 1, pch = 16,
     cex.axis = 1, cex.lab = 1, mgp = c(2,1,0), xlim = c(0, lim/6), ylim = c(0, lim), bty = "l",
     xlab = "Expected -log10 p-value", ylab = "Observed -log10 p-value")
legend("right", pch=16, cex=1, col=c("blue", "grey"),
       legend=c("Causal SNPs with PCs","Causal SNPs without PCs"), box.lty=1, bg="transparent")
abline(coef=c(0,1))
```

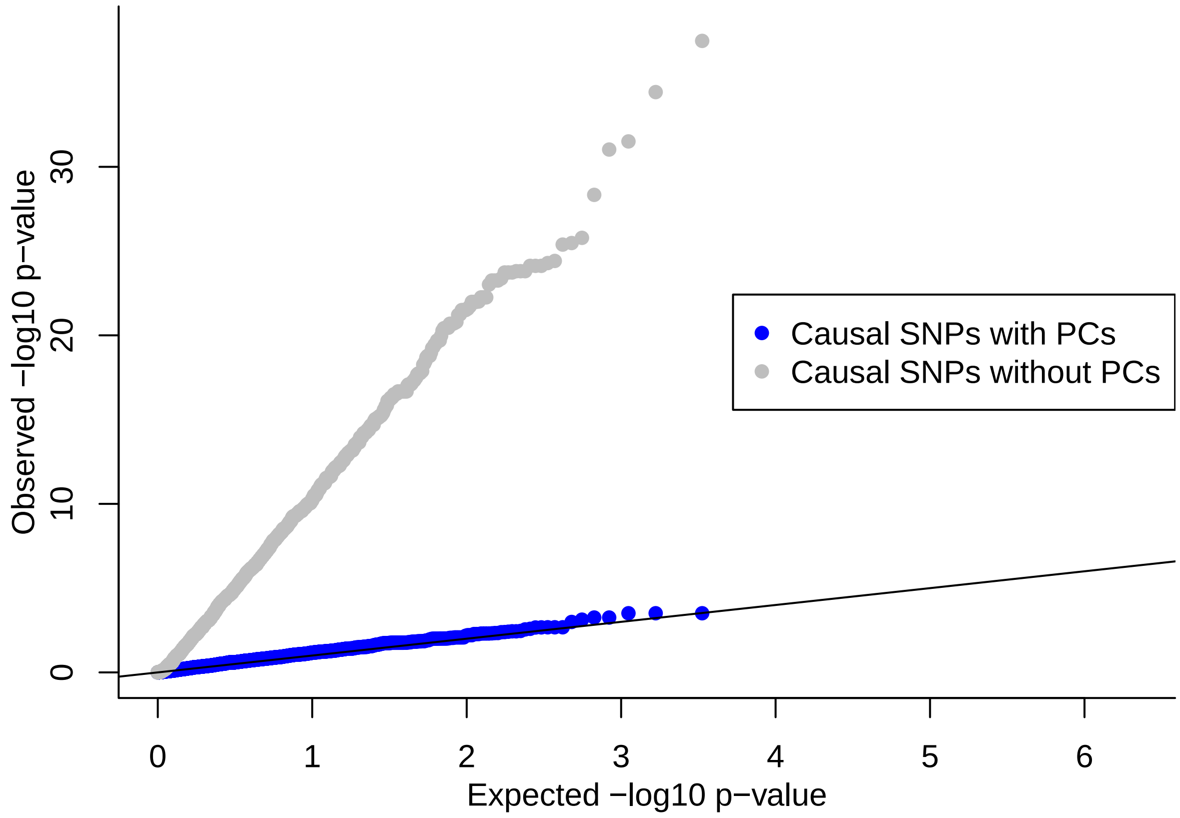

Case/Control Design for Rare Variants

Using chromosomes 19, 20, 21, and 22, 1,000 individuals were simulated each for cases and controls, where their target admixture set-up was the same as that used previously for common variants. Every fifth SNP was used. The prevalences were 0.2, 0.1, and 0.3 for CEU, CHB, and YRI, respectively. The mean effect size for rare variants was set at 0.01, under a Madsen-Browning weighting scheme.

```
chr_vec <- 19:22
indir <- "D:/HAP-SAMPLE2/"
dir.create(file.path(indir,"sampleChromoRare"))
```

```
## Warning in dir.create(file.path(indir, "sampleChromoRare")):
## 'D:\HAP-SAMPLE2\\sampleChromoRare' already exists
```

```
outdir <- "D:/HAP-SAMPLE2/sampleChromoRare/"
file.copy(from=paste0(indir,sample_filename), to=outdir)
for (i in chr_vec) { file.copy(from=paste0(indir,"genetic_map_chr",i,"_combined_b37.txt"), to=outdir) }
indir <- "D:/HAP-SAMPLE2/processed/"
sampleChromo_W(chr_vec, indir, outdir, sample_filename, retain_every=5, retain_snp=NULL,
               maf_threshold=0, n_cores=n_cores)
dir <- "D:/HAP-SAMPLE2/sampleChromoRare/"
loci_info_list <- makeLociInfo_W(chr_vec, dir)
loci_info <- loci_info_list$loci_info
chr_recomb <- loci_info_list$chr_recomb
snp <- selectSNP(loci_info)
ChrAlleleFreqs <- calcChrAlleleFreqs_W(snp, loci_info, chr_vec, dir, sample_filename,
                                       n_cores=n_cores, elig_pops)
sample_ma_list <- ChrAlleleFreqs$sample_ma_list
snp_map <- ChrAlleleFreqs$snp_map
AlleleFreq_by_pop <- calcAlleleFreq_by_pop(sample_ma_list, snp_map)
scoreRV_haps_out <- scoreRV_haps(dir, chr_vec, sample_filename, maf_thresh=0.01, elig_pops,
                                 mean_effect=0.01, weight_type="MB", n_cores=n_cores)
ind_probs <- solveRV_B0_W(spec_val=spec_prev, ind_scores=scoreRV_haps_out$ind_scores,
                          score_col="total", type="bin", lower=-100, upper=100)
B0Est <- ind_probs[match(elig_pops, ind_probs[, "pop"]), "B0Est"]
names(B0Est) <- elig_pops
rvSharesOut <- calcRV_shares_cc(mean_effect=0.01, ind_probs, hap_scores=scoreRV_haps_out$hap_scores,
                                score_col="total", prop_weighted=T)
caseSelectProbs <- rvSharesOut[,c("hap_tag","case_share")]
contSelectProbs <- rvSharesOut[,c("hap_tag","cont_share")]
colnames(caseSelectProbs) <- c("hap_tag","weight")
colnames(contSelectProbs) <- c("hap_tag","weight")
rvCase_genos <- matrix(0, nrow=nrow(adm_mat), ncol=length(snp))
colnames(rvCase_genos) <- snp
rvCont_genos <- rvCase_genos
dis_chr <- rep("no",length(chr_vec))
rvCase_cGM <- createGenoMult(dis_chr, dir, loci_info, sample_ma_list, adm_mat=adm_mat,
                             start_genos=rvCase_genos, snp_map, chr_recomb,
                             hap_select_probs=caseSelectProbs, prefix="rvCase", write_genos=T,
                             return_genos=F, write_every=1, n_cores=n_cores, compress=F)
rvCont_cGM <- createGenoMult(dis_chr, dir, loci_info, sample_ma_list, adm_mat=adm_mat,
                             start_genos=rvCont_genos, snp_map, chr_recomb,
                             hap_select_probs=contSelectProbs,prefix="rvCont", write_genos=T,
                             return_genos=F, write_every=1, n_cores=n_cores, compress=F)
```

Compare the probabilities of being a case, calculated post-simulation for 1,000 case and 1,000 control individuals, by admixture group and case/control status. Cases have higher scores than the controls. This would be expected, as the specified mean effect size was positive. The admixed groups generally fall in between their prescribed ancestral groups.

```
caseScores <- scoreRV_genos(dir, file_list=rvCase_cGM$fileList,
                            weights_adj_list=scoreRV_haps_out$weights_adj_list,
                            freq_list=scoreRV_haps_out$freq_list, adm_mat=adm_mat, B0Est=B0Est,
                            type="bin", n_cores=n_cores)
contScores <- scoreRV_genos(dir, file_list=rvCont_cGM$fileList,
                            weights_adj_list=scoreRV_haps_out$weights_adj_list,
                            freq_list=scoreRV_haps_out$freq_list, adm_mat=adm_mat, B0Est=B0Est,
                            type="bin", n_cores=n_cores)
caseScores <- as.data.frame(caseScores, stringsAsFactors=F)
contScores <- as.data.frame(contScores, stringsAsFactors=F)
caseScores$group <- group
contScores$group <- group
caseScoreAgg <- aggregate(score ~ group, caseScores, mean)
contScoreAgg <- aggregate(score ~ group, contScores, mean)
scoreComp <- cbind(caseScoreAgg, desc, contScoreAgg$score)
colnames(scoreComp) <- c("Group", "Case", "Desc.", "Cont.")
scoreComp <- scoreComp[, c("Group", "Desc.", "Case", "Cont.")]
scoreComp$Case <- round(scoreComp$Case, 2)
scoreComp$Cont. <- round(scoreComp$Cont., 2)
print(scoreComp)
```

```
##   Group       Desc. Case Cont.
## 1     1         CEU 0.28  0.14
## 2     2         CHB 0.14  0.07
## 3     3         YRI 0.52  0.16
## 4     4     CEU/CHB 0.20  0.10
## 5     5     CEU/YRI 0.39  0.15
## 6     6     CHB/YRI 0.29  0.10
## 7     7 CEU/CHB/YRI 0.29  0.12
## 8     8   Dirichlet 0.29  0.12
```

Quantitative Phenotype for Rare Variants

2,000 individuals were simulated for a quantitative trait phenotype, with the same target admixture set-up as the common variants. Every fifth SNP was used from chromosomes 19, 20, 21, and 22. The specified mean values for each ancestral population were 100, 105, and 85 for CEU, CHB, and YRI, respectively, with a standard deviation of 15. The mean effect size was set at 0.01, under a Madsen-Browning weighting scheme.

```
ind_probs <- solveRV_B0_W(spec_val=mu_vec, ind_scores=scoreRV_haps_out$ind_scores, score_col="total",
                          type="quant", lower=0, upper=100)
B0Est <- ind_probs[match(elig_pops, ind_probs[, "pop"]), "B0Est"]
names(B0Est) <- elig_pops
rvQuant_genos <- matrix(0, nrow=nrow(adm_mat), ncol=length(snp))
colnames(rvQuant_genos) <- snp
rvQuant_cGM <- createGenoMult(dis_chr, dir, loci_info, sample_ma_list, adm_mat=quant_adm_mat,
                              start_genos=rvQuant_genos, snp_map, chr_recomb, hap_select_probs=NULL,
                              prefix="rvQuant", write_genos=T, return_genos=F, write_every=1,
```

```
                                 n_cores=n_cores, compress=F)
quantScores <- scoreRV_genos(dir, file_list=rvQuant_cGM$fileList,
                             weights_adj_list=scoreRV_haps_out$weights_adj_list,
                             freq_list=scoreRV_haps_out$freq_list, adm_mat=quant_adm_mat, B0Est=B0Est,
                             type="quant", n_cores=n_cores)
```

Then compare the mean quantitative traits for 2,000 individuals by group. The non-admixed groups (groups 1-3) are generally close to the specified target means for those ancestral populations. The mean phenotype scores for the admixed groups are approximately between their ancestral populations.

```
quantPhenos <- quantScores + rnorm(nrow(quant_adm_mat), 0, sd)
quantPhenos <- as.data.frame(quantPhenos, stringsAsFactors=F)
quantPhenos$group <- group
quantPhenoAgg <- aggregate(score ~ group, quantPhenos, mean)
phenoComp <- cbind(quantPhenoAgg, desc)
colnames(phenoComp) <- c("Group", "Score", "Desc.")
phenoComp <- phenoComp[, c("Group", "Desc.", "Score")]
phenoComp$Score <- round(phenoComp$Score, 2)
print(phenoComp)
```

```
##   Group       Desc.  Score
## 1     1         CEU 100.98
## 2     2         CHB 101.65
## 3     3         YRI  84.77
## 4     4     CEU/CHB  98.32
## 5     5     CEU/YRI  91.77
## 6     6     CHB/YRI  94.41
## 7     7 CEU/CHB/YRI  95.41
## 8     8   Dirichlet  95.37
```
