# Supplementary material for: HAP-SAMPLE2: data-based resampling for association studies with admixture
Source: Bioinformatics. 2025 Jun 13;41(6):btaf333. doi: 10.1093/bioinformatics/btaf333 (PMC12208071; doi:10.1093/bioinformatics/btaf333)
Supplement: btaf333_Supplementary_Data [file btaf333_supplementary_data.zip › HAP_SAMPLE2_Supplementary file.pdf]

Supplementary File for HAP-SAMPLE2

George Sun[1], Bryan W. Ting[1], Fred A. Wright[2], and Yi-Hui Zhou*[2]

[1]Bioinformatics Research Center, North Carolina State University, Raleigh, NC, 27607, US

[2]Departments of Biological Sciences and Statistics, North Carolina State University, Raleigh, NC, 27607, US

April 4, 2025

1 Method

1.1 Case/Control Design for Common Variants

Recall that, according to the logistic regression model, we have as an individual's probability of disease: $\ln(\frac{\pi_i}{1-\pi_i}) = \boldsymbol{a_i^T}\boldsymbol{\alpha_s} + g_{is}\beta_s$. That is, each individual has a probability of disease given ancestry and genotype. However, in HAP-SAMPLE2, the actual sampling requires an Absolute Genotype specification—the probability of genotype given disease and ancestry—as simulated individuals have pre-specified disease statuses and user-provided ancestry proportions.

The HAPSAMPLE-2 approach enables multi-locus disease modeling, but it may not be obvious why the ancestry coefficients vary by locus. In order to effectively simulate the linkage disequilibrium patterns and artificial crossover processes, we solve for the necessary conditional probabilities for each causal locus at a time, marginalizing over other effects (recalling that at most one casual locus exists per chromosome). For illustration, let us focus on two subpopulations, for which the user specifies the marginal probabilities of disease. Suppose we have disease probability $p_1 = 0.2$ for the CEU subpopulation, and $p_2 = 0.1$ for CHB. When examining a locus that is not causal, and not linked to a causal locus, the intercept in the logistic model should be $\log(p_k/(1-p_k))$. But if a locus is causal, the genotype effects at the locus must be subtracted from the remaining marginal effects, according to the genotype frequencies in each population. Thus we must calculate the intercept values per population such that the overall disease probabilities are respected.

Figure S1 shows the intercept values among cases for these two populations across the 22 loci (one per autosome) shown for the case-control vignette, with

*To whom correspondence should be addressed: yihui_zhou@ncsu.edu

effect sizes that are log odds ratios of -0.1, 0, or 0.1 (roughly odds ratios ranging from 0.9 to 1.1). At causal loci with zero effect sizes, the intercept is indeed $\ln(p_k/(1-p_k))$. For loci with positive effect sizes, the intercepts must be lower to compensate. The precise values also vary according to the genotype frequencies within each population. Note that the actual logistic *modeling* performed by researchers will typically estimate intercept offset values that vary according to the case-control ratios, and we have accordingly worked out the conditional probabilities $P(D_i = 1|g_{is}, \mathbf{a}_i)$, also as shown below.

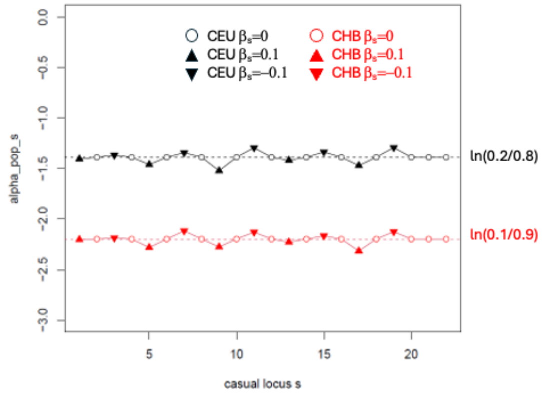

Figure S1: Hypothetical α_s values for two populations, using modest locus effect sizes.

For the modest but reasonable effect sizes here, these population-specific intercepts do not vary much, but the literature contains numerous examples of true loci where these differences are appreciable. HAPSAMPLE2 ultimately uses genotype frequencies among cases and controls, and computing these conditional probabilities accurately requires these calculations.

Using each individual's probability of being a case given genotype and ancestry, $P(D_i = 1|g_{is}, \boldsymbol{a_i})$, we can derive the probability of genotype given disease and ancestry, $P(g_{is}|D_i = 1, \boldsymbol{a_i})$. Following the logistic regression model:

$$\pi_i = P(D_i = 1|g_{is}, \boldsymbol{a_i})$$

$$\ln(\frac{P(D_i = 1|g_{is}, \boldsymbol{a_i})}{1 - P(D_i = 1|g_{is}, \boldsymbol{a_i})}) = \boldsymbol{a_i}^T \boldsymbol{\alpha_s} + g_{is}\beta_s = z_i$$

$$P(D_i = 1|g_{is}, \boldsymbol{a_i}) = \frac{P(D_i = 1, g_{is}, \boldsymbol{a_i})}{P(g_{is}|\boldsymbol{a_i})P(\boldsymbol{a_i})}$$

$$P(g_{is}|D_i = 1, \boldsymbol{a_i}) = \frac{P(D_i = 1|g_{is}, \boldsymbol{a_i})P(g_{is}|\boldsymbol{a_i})}{P(D_i = 1|\boldsymbol{a_i})} =$$

$$\frac{exp(z_i)P(g_{is}|\boldsymbol{a_i})}{(1 + exp(z_i))P(D_i = 1|\boldsymbol{a_i})}$$

Note that $P(D_i = 1|\boldsymbol{a_i})$ can be estimated from the user input for disease prevalence and a given individual's ancestry proportions, and $P(g_{is}|\boldsymbol{a_i})$ can be calculated from the data.

Absolute genotype values for the controls can be obtained via a similar formulation as that of cases. Recalling the last equation previously from cases and substituting in $D_i = 0$ for $D_i = 1$:

$$P(D_i = 0|g_{is}, \boldsymbol{a_i}) = \frac{P(D_i = 0|g_i s, \boldsymbol{a_i})P(g_{is}|\boldsymbol{a_i})}{P(D_i = 0|\boldsymbol{a_i})} =$$

$$\frac{(1 - P(D_i = 1|g_{is}, \boldsymbol{a_i}))P(g_{is}|\boldsymbol{a_i})}{1 - P(D_i = 1|\boldsymbol{a_i})} =$$

$$\left(1 - \frac{e^{z_i}}{1 + e^{z_i}}\right)\left(\frac{P(g_{is}|\boldsymbol{a_i})}{1 - P(D_i = 1|\boldsymbol{a_i})}\right)$$

Figure S2 shows the simulation process for haplotypes, conditional on the causal SNP, as previously described (Wright *et al.*, 2007).

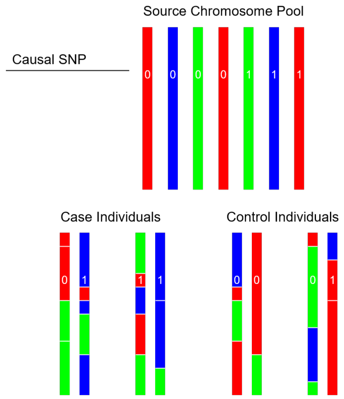

Figure S2: Stylized illustration of simulating chromosomes for cases and controls with common variants, using three ancestral populations. A positive effect size is assumed for the alternative allele, thus the alternative allele is found with higher frequency in the two case individuals than in the two control individuals. White borders in the simulated chromosomes represent locations where crossovers occurred.

2 Example Simulations

Example simulations were performed using data from 1000 Genomes (Auton, A. and Abecasis, G. and Altshuler, D. et al., 2015; IGSR: The International Genome Sample Resource, 2015) to illustrate various aspects of the functionalities of HAP-SAMPLE2.

2.1 Case/Control Design for Common Variants

1,000 cases and 1,000 controls were simulated. Every fifth locus was used to form the simulated haplotypes and genotypes; a minor allele threshold of 0.02 was used.

A disease prevalence was set at 0.2, 0.1, and 0.3 for CEU, CHB, and YRI, respectively. For cases, 300 individuals were designated to have target admixture proportions of 100%, 100 each for CEU, CHB, and YRI. 300 individuals were targeted to have 50% admixture proportions, 100 for each pairwise combination between CEU, CHB, and YRI. 100 individuals were designated to have 1/3 admixture between the three. 300 individuals had target admixture proportions generated from the Dirichlet distribution with means of 1/3. Target admixture proportions for the controls were specified the same way.

22 chromosomes were used, with effect sizes of $\{1, 0, -1, 0\}$ for the first 20, and $\{0, 0\}$ for the last two chromosomes. This is shown in Supplementary Table S1, along with the mean allele counts for case and control individuals tabulated post-simulation. As expected, for SNPs with positive effect sizes, cases had noticeably higher mean counts than that of controls. For SNPs with negative effect sizes, controls analogously had higher mean counts than that of cases. When the effect sizes for SNPs were 0, the mean counts were similar between that of cases and controls.

The estimated admixture proportions for cases, calculated from the simulated genotypes, are shown in Supplementary Figure S3. Controls looked very similar. Function `sNMF` from R package `LEA`, which provides `STRUCTURE`-like functionality for admixture, was used to calculate the estimated admixture proportions (Francois, 2016; Frichot and Francois, 2014).

The admixture calculations were done in unsupervised fashion—i.e. no reference populations were designated as the three ancestral populations. The mean estimated admixture proportions by group indeed reflected the target admixture proportions.

PCA plots were also constructed using 1,500 loci randomly sampled from each of the 22 chromosomes, as another way to look at admixture results across groups. The first two principal components for cases are shown in Supplementary Figure S4. The individuals with targeted proportions of 100% of the ancestral populations indeed tightly cluster amongst themselves and comprise the nodes of a triangle, and the individuals with 50% of two populations form the edges. The individuals with targeted proportions of $\frac{1}{3}$ each are found in the center of the triangle, and the Dirichlet-targeted individuals are scattered across the triangle. PCA results for controls looked largely the same.

Supplementary Figure S5 depicts the 300 case individuals with Dirichlet-generated admixture proportions. Each plot compares targeted versus realized admixture for a given ancestral source population. The targeted ratios were derived using the Dirichlet distribution, and `sNMF` provided the recognized admixture proportions.

As a further illustration, we show here the net effect of the case-control

Table S1: Comparison of mean alternative allele counts between 1,000 simulated case and 1,000 simulated control individuals.

Chr.	SNP	Effect Size	Cases	Controls
1	rs72638544	1	0.22	0.07
2	rs7579608	0	0.19	0.20
3	rs10510310	-1	0.09	0.22
4	rs7685911	0	0.38	0.34
5	rs1901498	1	1.34	0.87
6	rs4960131	0	1.02	1.05
7	rs10237521	-1	0.90	1.38
8	rs4433167	0	1.30	1.29
9	rs7865441	1	1.21	0.78
10	rs4584478	0	0.53	0.53
11	rs9804657	-1	0.51	0.94
12	rs55768228	0	1.48	1.40
13	rs7991629	1	0.55	0.26
14	rs28721413	0	1.22	1.22
15	rs7180505	-1	1.08	1.52
16	rs9935770	0	0.79	0.84
17	rs11247560	1	1.34	0.89
18	rs62078854	0	0.49	0.49
19	rs12980335	-1	0.50	0.97
20	rs156338	0	1.24	1.23
21	rs9980561	0	0.72	0.81
22	rs5746362	0	1.29	1.32

sampling, with estimation of odds ratios and power that have the expected behavior, for $|\beta_s| \leq 1.0$. We follow the example in the previous paragraphs (i.e. proportions and admixture scheme for CEU, CHB. and YRI), with the same population-specific probabilities of disease. To accord with modern sampling designs, we performs simulations with 10,000 cases and 10,000 controls for each simulation. Three SNPs were chosen for illustration, with minor allele frequencies (determined for a population with 1/3 mixture of each of CEU, CHB, and YRI) covering the range 0.1-0.5. The chosen SNPs were rs7579608 (chr 2, MAF=0.1), rs1901498 (chr 5, MAF=0.5), and rs62078854 (chr 18, MAF=0.25). The power and sampling characteristics are primarily determined by the MAF. 1,000 simulations were performed for each locus for each choice of β_s ranging from -1.0 to 1.0 in increments of 0.05. For each simulation, logistic regression analysis was performed using logistic regression (`glm` in R) using the admixture predictors and the SNP genotype (0, 1, or 2). The β coefficient estimate and standard error was computed, and 90% and 95% confidence intervals calculated,

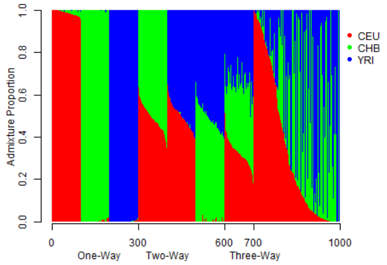

Figure S3: Admixture plots for case individuals. "One-Way" refers to individuals with target admixture proportions of 1 for a given population. "Two-Way" refers to individuals with target admixture proportions of $\frac{1}{2}$ for each of two populations, and likewise "Three-Way" for three populations of $\frac{1}{3}$ each. 1,500 loci were randomly sampled from each of the chromosomes. The admixture proportions were calculated in an unsupervised fashion by function `sNMF` from R package `LEA` (Francois, 2016; Frichot and Francois, 2014)

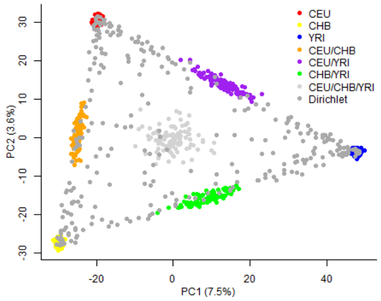

Figure S4: PCA plot for 1,000 case individuals, using 22 chromosomes. 1,500 loci were randomly sampled from each of the chromosomes to calculate the principal components.

and $z = \hat{\beta}/SE$ used to provide two-sided p-values.

The results of these analyses are shown in Supplementary Figure S6, showing unbiased estimation (panel A), correct confidence coverage at the 90% and 95% levels (panel B), and All of the behavior shown is expected, and power to detect the locus as β varies (panel C).

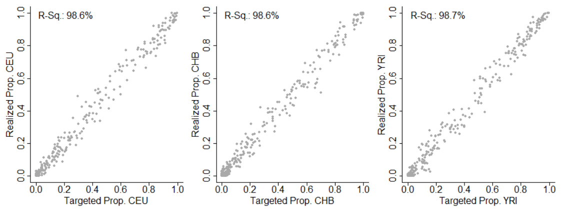

Figure S5: Targeted vs. realized admixture proportions for the 300 case individuals with Dirichlet-drawn targeted admixture proportions, by the three ancestral source populations.

2.2 Quantitative Phenotype for Common Variants

Using a similar admixture set-up as for the case/control design, 2,000 individuals were simulated for a quantitative phenotype. Loci rs12980335, rs156338, rs9980561, rs6516850, rs2838791, and rs5746362 were specified as the causal loci, with effect sizes of 5, 0, 10, 0, and 15, respectively. The specified standard deviation was 15. The specified population means for CEU, CHB, and YRI were 100, 105, and 85, respectively.

The Manhattan plot in Fig. S7 displays GWAS results, conducted upon the simulated genotypes and phenotypes, using the first four principal components as covariates. 2,000 SNPs were randomly chosen to compute the first four principal components. The case/control design for common variations illustrates the locations of causal SNPs with non-zero impact sizes at the window's peak. Similarly, the proximity of loci to the causal SNPs is positively associated with higher rates of LD.

The simulation for a quantitative phenotype with common variants was rerun, this time with the effect sizes for the SNPs being all 0 and the other settings left unchanged. The q-q plot in Supplementary Figure S8 illustrates that neglecting to account for population stratification can result in more false positives, as the grey line (corresponding to -log10 p-values of the GWAS without the first four PCs as covariates) diverges from the unit line much sooner than does the blue line (corresponding to -log10 p-values of the GWAS including the first four PCs as covariates).

References

Auton, A. and Abecasis, G. and Altshuler, D. et al. (2015). A global reference for human genetic variation. *Nature*, **526**, 68–74.

Francois, O. (2016). Running structure-like population genetic analyses with r. R Tutorials in Population Genetics, U. Grenoble-Alpes, pages 1-9.

Frichot, E. and Francois, O. (2014). LEA: an R pack-

age for Landscape and Ecological Association studies. https://www.bioconductor.org/packages/release/bioc/vignettes/LEA/inst/doc/LEA.pdf.

IGSR: The International Genome Sample Resource (2015). 1000 genomes release: Phase 3. Index of /vol1/ftp/release/20130502/ ftp://ftp.1000genomes.ebi.ac.uk/vol1/ftp/release/20130502/.

Wright, F. A. *et al.* (2007). Simulating association studies: a data-based resampling method for candidate regions or whole genome scans. *Bioinformatics*, **23**(19), 2581–2588.

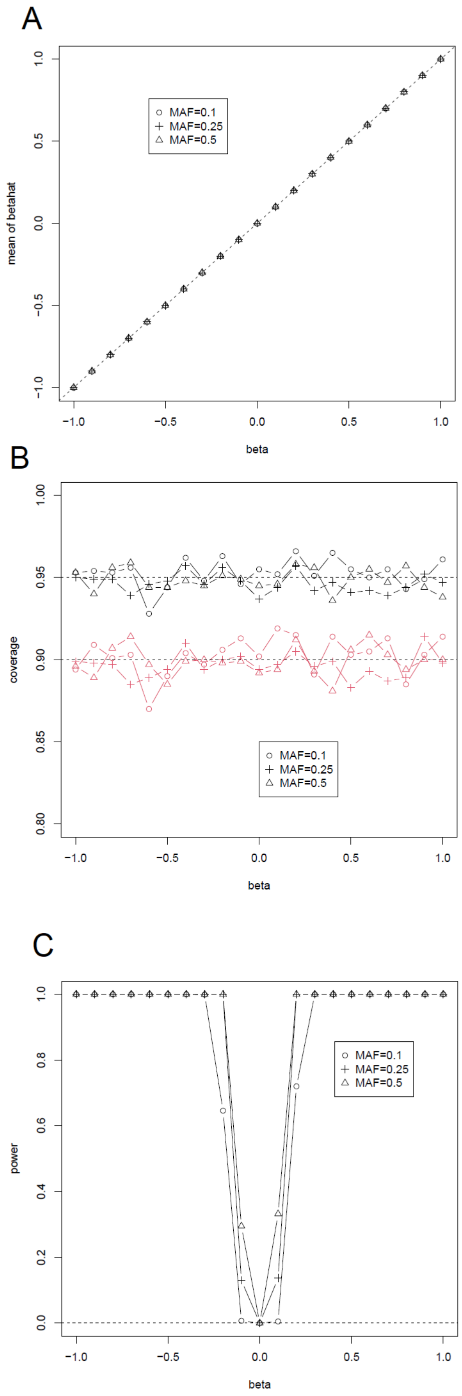

Figure S6: The case-control example. Panel A shows the mean of $\hat{\beta}$ values across simulations. For all MAF values, the estimates are unbiased. Panel B shows the confidence interval coverage for 90% and 95% intervals. For all MAF values, the coverage is correct (some sampling variation applies). Panel C shows power curves using significance level 5×10^{-8}, which show that the power for extreme β values is near 1, but also that the power increases with MAF.

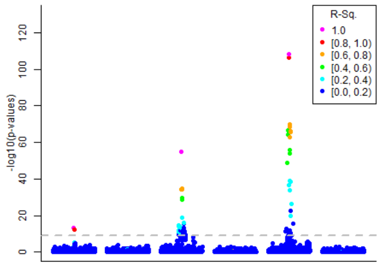

Figure S7: Manhattan plot of simulated GWAS results for the quantitative phenotype using common variants, with the first four principal components as covariates alongside the SNP values. The causal SNP is highlighted in magenta. The 1,000 loci closest to the causal SNP are depicted in each window. The grey dashed line corresponds to a Bonferroni-corrected p-value of 0.05, applied at the genome-wide level of 84.4 million. The R^2 values correspond to the degree of linkage disequilibrium for the loci with the nearest causal SNP.

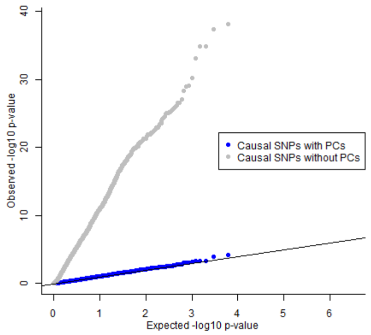

Figure S8: q-q plot of simulated GWAS results, comparing observed and expected -log10 p-values. The effect sizes for the SNPs were set at 0 (null hypothesis), so this example shows that standard ancestry control is needed, and is sufficient, for these data.
